# TDO2^+^ myofibroblasts mediate immune suppression in malignant transformation of squamous cell carcinoma

**DOI:** 10.1172/JCI157649

**Published:** 2022-10-03

**Authors:** Simeng Hu, Huanzi Lu, Wenqiang Xie, Dikan Wang, Zhongyan Shan, Xudong Xing, Xiang-Ming Wang, Juan Fang, Wei Dong, Wenxiao Dai, Junyi Guo, Yanshu Zhang, Shuqiong Wen, Xin-Yu Guo, Qianming Chen, Fan Bai, Zhi Wang

**Affiliations:** 1 Biomedical Pioneering Innovation Center (BIOPIC), School of Life Sciences, Academy for Advanced Interdisciplinary Studies (AAIS), and Peking University–Tsinghua University–National Institute of Biological Sciences Joint Graduate Program (PTN), Peking University, Beijing, China.; 2 Hospital of Stomatology, Guanghua School of Stomatology, Guangdong Provincial Key Laboratory of Stomatology, and Zhongshan School of Medicine, Sun Yat-Sen University, Guangzhou, Guangdong, China.; 3 Zhongshan School of Medicine, Sun Yat-sen University, Guangzhou, Guangdong, China.; 4 The First School of Clinical Medicine, Southern Medical University, Guangzhou, Guangdong, China.; 5 Hospital of Stomatology, and Key Laboratory of Oral Biomedical Research of Zhejiang Province, School of Stomatology, Zhejiang University School of Medicine, Hangzhou, Zhejiang, China.; 6 Beijing Advanced Innovation Center for Genomics (ICG), and; 7 Center for Translational Cancer Research, First Hospital, Peking University, Beijing, China.

**Keywords:** Immunology, Oncology, Cancer immunotherapy

## Abstract

Characterization of the dynamic change in the immunological landscape during malignant transformation from precancerous lesions to cancerous lesions in squamous cell carcinoma (SCC) is critical for the application of immunotherapy. Here, we performed single-cell RNA-Seq (scRNA-Seq) of 131,702 cells from 13 cancerous tissues of oral squamous cell carcinoma (OSCC), 3 samples of precancerous oral leukoplakia, and 8 adjacent normal samples. We found that tumor-infiltrating CD4^+^ and CD8^+^ T cells were functionally inhibited by immunosuppressive ligands expressed on various types of myeloid cells or neutrophils in the process of oral carcinogenesis. Notably, we identified a subset of myofibroblasts that exclusively expressed tryptophan 2,3-dioxygenase (TDO2). These TDO2^+^ myofibroblasts were located distally from tumor nests, and both CD4^+^ and CD8^+^ T cells were enriched around them. Functional experiments revealed that TDO2^+^ myofibroblasts were more likely to possess the ability for chemotaxis toward T cells but induced the transformation of CD4^+^ T cells into Tregs and caused CD8^+^ T cell dysfunction. We further showed that use of the TDO2 inhibitor LM10 attenuated the inhibitory states of T cells, restored the T cell antitumor response, and prevented the progression of OSCC malignant transformation in murine models. Our study reveals a multistep transcriptomic landscape of OSCC and demonstrates that TDO2^+^ myofibroblasts are potential targets for immunotherapy.

## Introduction

Oral squamous cell carcinoma (OSCC), a malignancy that occurs in the epithelium of the oral cavity, is the predominant type of head and neck squamous cell carcinoma (HNSCC). It accounts for 1.8% of newly diagnosed cancer cases annually worldwide (1). The main causes of OSCC are cigarette smoking, alcoholic consumption, and betel nut chewing (2). Although OSCC treatment has evolved from surgical resection to multidisciplinary treatments, including surgery, radiotherapy, and chemotherapy, the 5-year overall survival (OS) rate remains approximately 50%. Oral carcinogenesis is a multistage process that initiates from oral precancerous lesions, of which oral leukoplakia (OLK) is the most common type (3). A recent cohort study revealed that the malignant transformation rate of OLK is approximately 11.7%–23.1% (4). Interventions need to be delivered during the early stage of OSCC to prevent its progression and achieve a better prognosis for patients with OSCC.

In the past decade, immune checkpoint blockade (ICB), which targets inhibitory receptors on T cells, including programmed cell death 1 (PD-1) and cytotoxic T lymphocyte–associated protein 4 (CTLA4), has achieved great success in the treatment of melanoma, non–small cell lung cancer, and renal cell carcinoma (5). Major (>90%) or complete responses have been seen in a fraction of patients with HNSCC (6); however, less than 20% of patients with HNSCC or OSCC showed a clinical response to ICB (6–9), demonstrating that most patients cannot benefit from current immunotherapies and that new immune oncologic approaches may provide significant benefit to patients. The tumor microenvironment (TME) is a complex ecosystem composed of tumor cells, T cells, B cells, myeloid cells, and stromal cells. To improve the efficacy of immunotherapy for patients with OSCC, the cell type distribution and the dynamic changes in the TME during the multistep malignant transformation process of oral carcinogenesis need to be characterized.

Myofibroblasts that express α–smooth muscle actin (α-SMA) are the major stromal cells in the TME (10). Myofibroblasts are derived from several types of cells, including normal fibroblasts, adipose-derived stem cells (ADSCs), and pericytes (11, 12). The functions of myofibroblasts have been widely described, and myofibroblasts have been shown to participate in tumor proliferation and invasion, angiogenesis, and metastasis (13–15). However, the role of myofibroblasts in immune regulation remains largely unknown. Recent studies have reported that myofibroblasts in the TME of pancreatic adenocarcinoma mediate immune suppression by TGF-β signaling (16, 17), and myofibroblasts in the TME of breast cancer promote an immunosuppressive microenvironment by attracting and retaining Tregs (18). However, additional mechanisms by which myofibroblasts mediate immune suppression have not been reported.

In the present study, we systematically profiled and characterized the heterogeneity, differentiation states, and interactions of immune cells and stromal cells isolated from 24 fresh surgically dissected samples, including OSCC, OLK, and adjacent normal tissues, using single-cell RNA-Seq (scRNA-Seq). By comparing the relative proportions of cells in OSCC, OLK, and normal tissues, we found that CD4^+^ and CD8^+^ T cells underwent a significant cell fate transition from a naive state to an exhausted state during the process of oral carcinogenesis. In addition, we characterized the dynamic changes in myeloid cells, neutrophils, and stromal cells during the process of oral carcinogenesis. Notably, we discovered a subset of myofibroblasts expressing tryptophan 2,3-dioxygenase (TDO2), which played a critical role in T cell sequestration and suppression. Moreover, we confirmed that the TDO2 inhibitor LM10 could reverse the inhibitory state of T cells and prevent the progression of OSCC in murine models. Our findings reveal an immunosuppressive mechanism by which stromal cells regulate T cells and provide a potential therapeutic target for OSCC.

## Results

### Single-cell transcriptomic landscape of precancerous and cancerous tissues in oral carcinogenesis.

**Figure 1:**
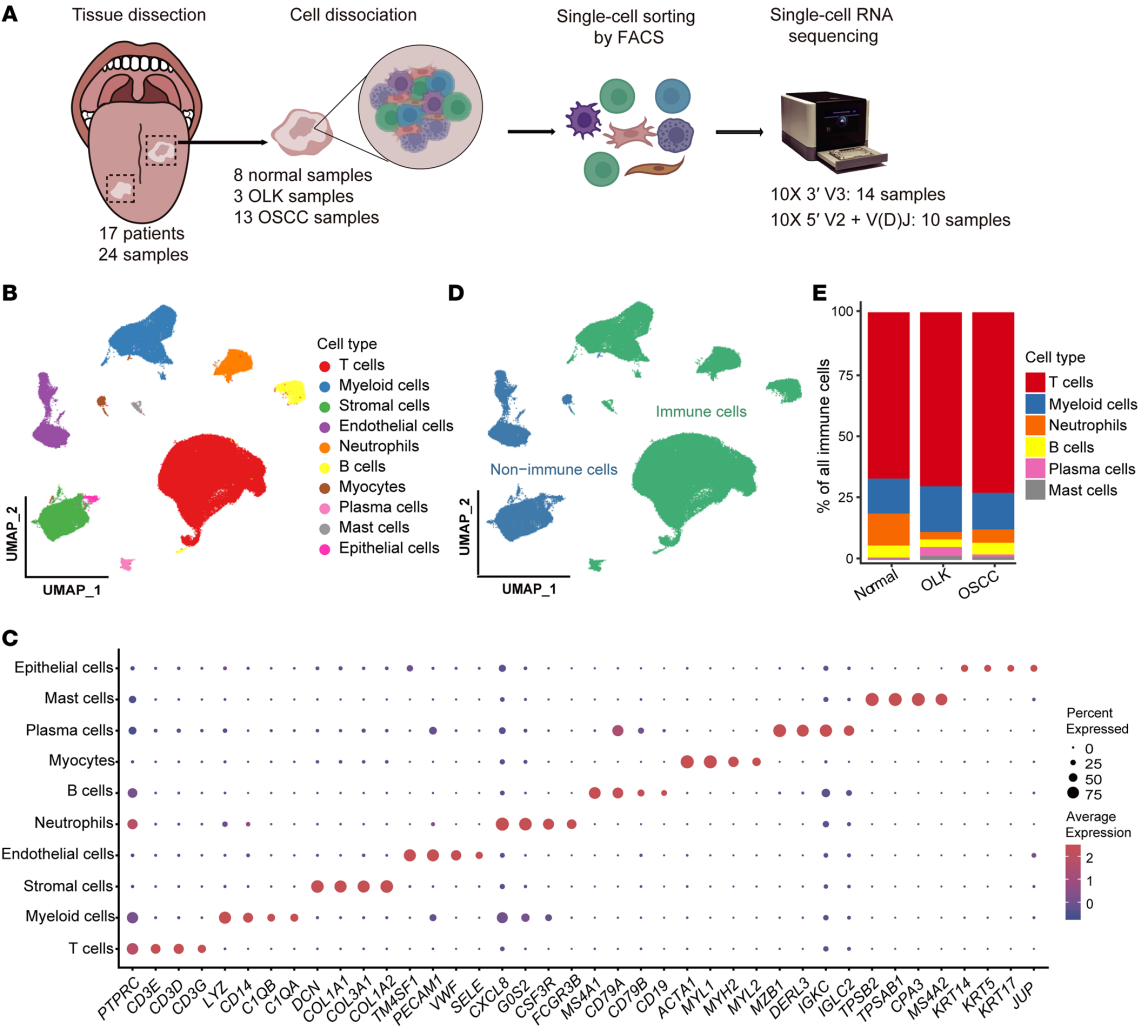
Single-cell transcriptomic landscape of adjacent normal, OLK, and OSCC tissues. (**A**) Overview of the workflow and the experimental design for scRNA-Seq. (**B**) UMAP plot showing the clustering results of 10 major cell types for 131,702 high-quality single cells from adjacent normal, OLK, and OSCC tissues. The colors represent the major cell types. (**C**) Dot plot showing the highly expressed marker genes in each major cell type. The dot size represents the percentage of cells expressing the marker genes in each major cell type, and the dot color represents the average expression level of the marker genes in each cell type. Red indicates high expression, and blue indicates low expression. (**D**) UMAP plot showing the distribution of immune and nonimmune cells among all cells. Green indicates immune cells, and blue indicates nonimmune cells. (**E**) Stacked histogram showing the percentages of immune cell types among total immune cells from adjacent normal, OLK, and OSCC tissues.

To systematically survey the cellular diversity of malignant transformation during oral carcinogenesis, we performed droplet-based scRNA-Seq (10X Genomics) of 13 OSCC samples, 3 OLK samples, and 8 adjacent normal samples (Figure 1A and Supplemental Table 1; supplemental material available online with this article; https://doi.org/10.1172/JCI157649DS1). We used FACS to sort all live cells from dissociated single-cell suspensions. A single-cell 5′ reagent kit was coupled with single-cell V(D)J sequencing to obtain T cell receptor (TCR) clonotypes for 10 samples, and a single-cell 3′ reagent kit was used for 14 samples. By applying stringent quality control methods, we obtained 131,702 high-quality cells, including 72,268 cells (54.87%) from OSCC tissues, 24,470 cells (18.58%) from OLK tissues, and 34,964 cells (26.55%) from adjacent normal samples. After data preprocessing, sample integration, and principal component analysis (PCA), high-quality cells were partitioned into 10 major clusters (Figure 1B, Supplemental Figure 1, B–F, and Supplemental Table 2) by graph-based uniform manifold approximation and projection (UMAP). According to the expression of known canonical marker genes (19–22), we identified these 10 major clusters as immune cells (T cells, myeloid cells, neutrophils, B cells, plasma cells, and mast cells) and nonimmune cells (stromal cells, endothelial cells, myocytes, and epithelial cells) (Figure 1C and Supplemental Table 3). In total, 107,405 immune cells were captured, accounting for 81.55% of all cells (Figure 1D). This cell proportion was validated by FACS (Supplemental Figure 1A). The proportion of T cells in normal tissues (67.3%) was lower than that in OLK (70.4%) and OSCC (73%) tissues (Figure 1E and Supplemental Figure 1G). This finding indicates that the TME of OSCC is enriched with a large number of T cells, highlighting their major antitumor function, which is also consistent with previous studies of head and neck cancers (21, 22).

### T cell dysfunction and cell state transitions in precancerous and cancerous tissues in oral carcinogenesis.

**Figure 2:**
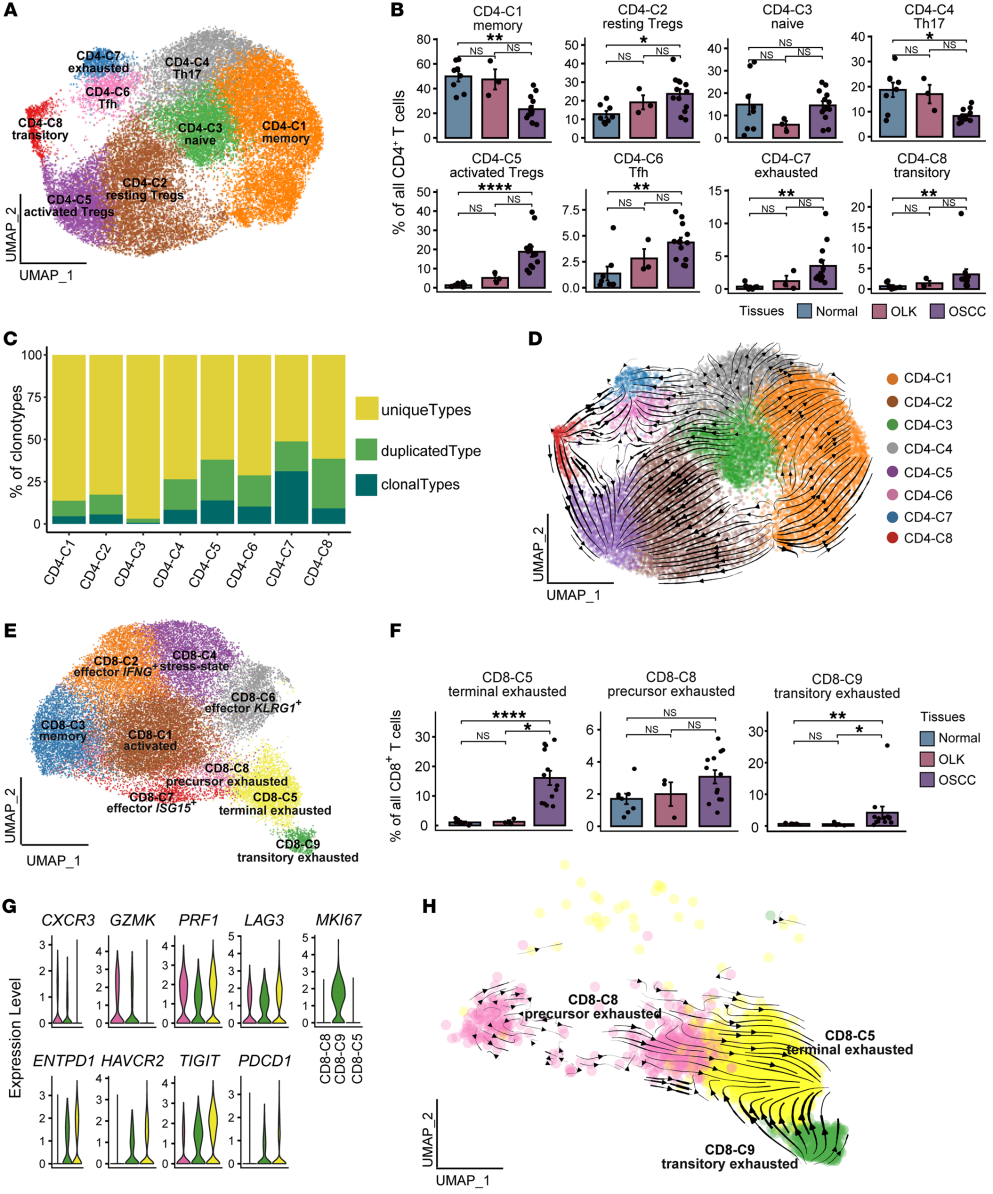
T cell dysfunction and cell state transitions in OSCC. (**A**) UMAP plot showing the distribution of CD4^+^ T cell subsets. Each color represents a CD4^+^ T cell subset. (**B**) Bar plots showing the percentage of CD4^+^ T cell subsets among total CD4^+^ T cells in adjacent normal, OLK, and OSCC tissues. Each color represents a tissue type. (**C**) Bar plots showing the percentages of TCR-expanded clonotypes in the CD4^+^ T cell subsets. The colors indicate different expanded clonotypes. (**D**) The RNA velocity of CD4^+^ T cells was visualized on the UMAP plot based on the stochastic model in the scVelo algorithm, suggesting a putative differentiation direction for CD4^+^ T cells. The arrows indicate the putative differentiation direction. (**E**) UMAP plot showing the distribution of CD8^+^ T cell subsets. Each color represents a CD8^+^ T cell subset. (**F**) Bar plots showing the percentages of 3 subsets of CD8^+^ T cells among total CD8^+^ T cells in adjacent normal, OLK, and OSCC tissues. (**G**) Violin plots showing the expression levels of effector molecules and immune-inhibitory receptors in 3 subsets of CD8^+^ T cells. (**H**) The RNA velocity of CD8^+^ T cell subsets was visualized on the UMAP plot based on the stochastic model in the scVelo algorithm, suggesting a putative differentiation direction for CD8^+^ T cell subsets. Arrows represent the putative differentiation direction. *P < 0.05, **P < 0.01, and ****P < 0.0001, by Kruskal-Wallis test followed by Bonferroni’s multiple-comparison test (**B** and **F**).

To investigate the cellular state and function of T cells in the TME, we clustered 66,724 T cells into sets of CD4^+^ T cells (*CD4*^+^), CD8^+^ T cells (*CD8A*^+^), NK T (NKT) cells (*KLRD1*^+^), and γδT cells (*TRDC*^+^) (Supplemental Figure 2, A and B, and Supplemental Table 3). CD4^+^ T cells were reclustered into sets of naive CD4^+^ T cells (CD4-C3; *CCR7^+^*), memory CD4^+^ T cells (CD4-C1; *IL7R^+^*), Th17 cells (CD4-C4; *IL17F^+^ IL17A^+^*), exhausted CD4^+^ T cells (CD4-C7; *LAG3^+^HAVCR2^+^*), follicular helper T (Tfh) cells (CD4-C6; *IL21^+^*), transitory CD4^+^ T cells (CD4-C8; *MKI67^+^*), resting Tregs (CD4-C2; *FOXP3^lo^CTLA4^lo^KLF2^+^*), and activated Tregs (CD4-C5; *FOXP3^hi^CTLA4^hi^TNFRSF4^+^*) (23, 24) (Figure 2A, Supplemental Figure 2C, and Supplemental Table 4).

We observed that the relative percentages of Tregs (CD4-C2 and CD4-C5), transitory CD4^+^ T cells (CD4-C8), exhausted CD4^+^ T cells (CD4-C7), and Tfh cells (CD4-C6) showed a gradual increase from normal to OLK to OSCC tissues (Figure 2B and Supplemental Table 5). In addition, exhausted CD4^+^ T cells (CD4-C7) and activated Tregs (CD4-C5) harbored a relatively high level of clonal expansion (Figure 2C). Furthermore, we used scVelo to infer the differentiation trajectory of CD4^+^ T cells (Figure 2D). The arrow represents the putative differentiation direction. Naive CD4^+^ T cells (CD4-C3) differentiated mainly into memory CD4^+^ T cells (CD4-C1), Th17 cells (CD4-C4), and resting Tregs (CD4-C2). Resting Tregs (CD4-C2) tended to differentiate into activated Tregs (CD4-C5). Transitory CD4^+^ T cells (CD4-C8) with a strong proliferative capacity differentiated into activated Tregs (CD4-C5) and into exhausted CD4^+^ T cells (CD4-C7) as well as Tfh cells (CD4-C6). These results suggest that, during the process of oral carcinogenesis, CD4^+^ T cells undergo cell state transition, differentiating into activated Tregs with highly immunosuppressive functions and highly exhausted CD4^+^ T cells.

Next, CD8^+^ T cells were classified into sets of activated CD8^+^ T cells (CD8-C1; *CRTAM^+^*), memory CD8^+^ T cells (CD8-C3; *IL7R^+^*), effector CD8^+^ T cells expressing IFN-γ (CD8-C2; *IFNG^+^*), effector CD8^+^ T cells responding to IFN (CD8-C7; *ISG15^+^*), effector CD8^+^ T cells (CD8-C6; *KLRG1^+^*), stress-state CD8^+^ T cells (CD8-C4; *HSPA1A^+^*), precursor exhausted CD8^+^ T cells (CD8-C8; *GZMK^+^CXCR3^+^*), transitory exhausted CD8^+^ T cells (CD8-C9; *MKI67^+^*), and terminally exhausted CD8^+^ T cells (CD8-C5; *ENTPD1^+^HAVCR2^+^*) (Figure 2E, Supplemental Figure 2D and Supplemental Table 6). The relative percentages of terminally exhausted CD8^+^ T cells (CD8-C5), precursor exhausted CD8^+^ T cells (CD8-C8), and transitory exhausted CD8^+^ T cells (CD8-C9) in OSCC tissues were higher than those of their OLK and normal counterparts (Figure 2F, Supplemental Figure 2F, and Supplemental Table 7). Although other CD8^+^ T cell subsets had relatively high levels of clonal expansion, terminally exhausted CD8^+^ T cells (CD8-C5) harbored the highest level of clonal expansion and expressed high levels of T cell suppressor molecules, including *TIGIT*, *ENTPD1*, *HAVCR2*, *LAG3*, and *PDCD1* (Figure 2G and Supplemental Figure 2E). The precursor exhausted signature score (25) of the CD8-C8 cells was significantly higher than those of the CD8-C9 and CD8-C5 cells (*P* < 0.0001), whereas the terminal exhaustion signature score was ranked as follows: CD8-C8 < CD8-C9 < CD8-C5 (Supplemental Figure 2G). This finding is consistent with a recent study (26) showing the presence of transitory proliferative CD8^+^ T cells during differentiation from precursor exhausted to terminally exhausted CD8^+^ T cells. In addition, we inferred the differentiation trajectory of CD8^+^ T cells using scVelo and found that the precursor exhausted and transitory exhausted CD8^+^ T cells switched into terminally exhausted CD8^+^ T cells (Figure 2H). These results suggest that there was a transition in the state of CD8^+^ T cells, indicating that CD8^+^ T cells were consistently converted to an exhausted state in the OSCC microenvironment.

### Immune-suppressive myeloid cells and neutrophils inhibit T cell function.

**Figure 3:**
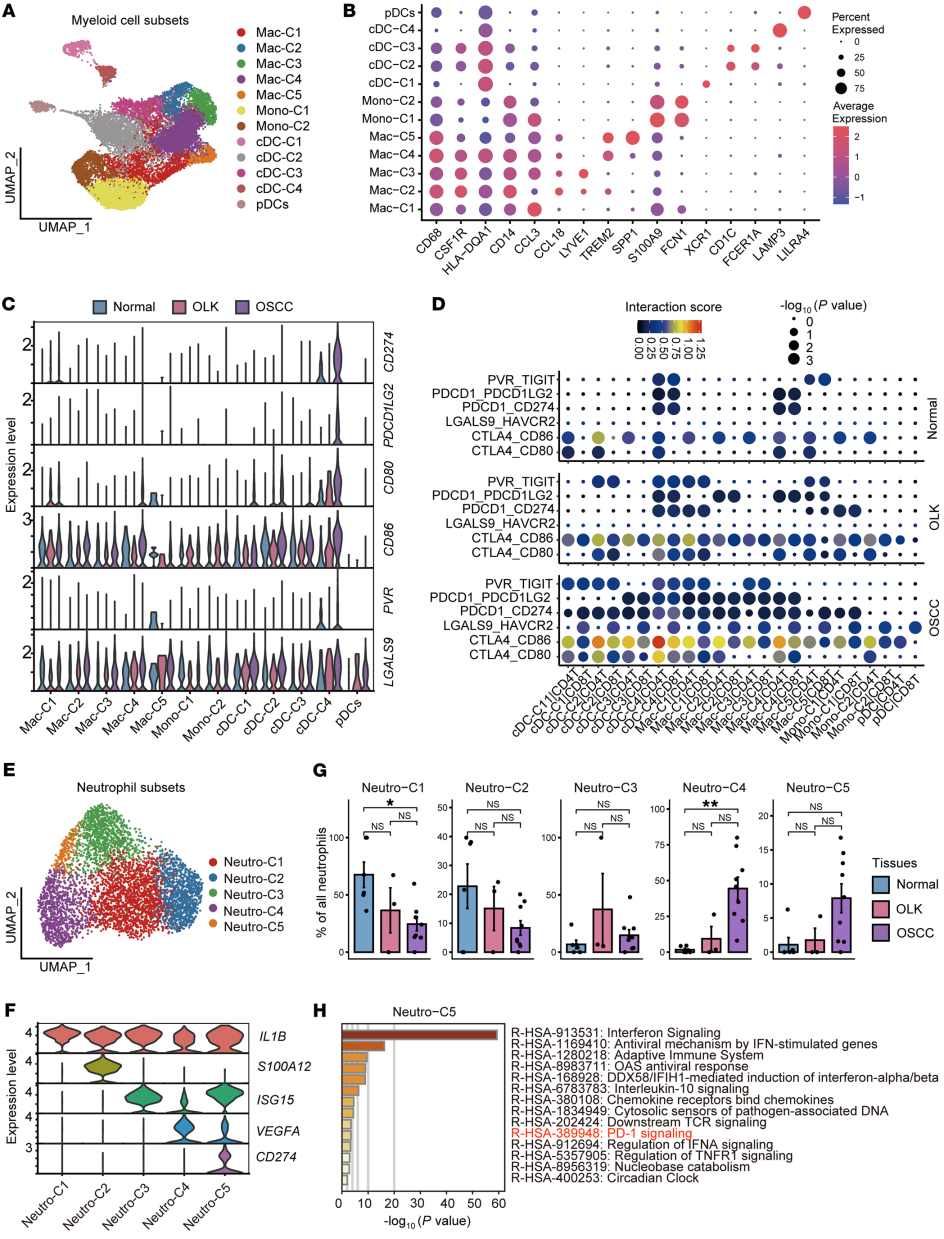
Subsets of myeloid cells and neutrophils that inhibit the function of T cells. (**A**) UMAP plot showing the distribution of myeloid cell subsets. Each color represents a subset of myeloid cells. (**B**) Dot plot showing highly expressed marker genes in myeloid cell subsets. (**C**) Violin plot showing the expression levels of immunosuppressive ligand molecules in myeloid cell subsets in each tissue. (**D**) Dot plot showing the interaction intensity between myeloid cell subsets and CD4^+^ and CD8^+^ T cells according to CellPhoneDB analysis. Blue indicates low-intensity interaction and red indicates high-intensity interaction. The dot size represents –log_10_ (*P* value), and a larger dot indicates a smaller *P* value. (**E**) UMAP plot showing the distribution of neutrophil subsets. Each color represents a subset of neutrophils. (**F**) Violin plot showing the expression levels of specifically expressed genes in neutrophil subsets. Each color represents a gene. (**G**) Bar plots showing the percentages of neutrophil subsets among the total neutrophils in adjacent normal, OLK, and OSCC tissues. *P < 0.05 and **P < 0.01, by Kruskal-Wallis test followed by Bonferroni’s multiple-comparison test. (**H**) Bar plot showing the results of enrichment analysis of the set of genes highly expressed in Neutro-C5 in the Reactome database, with the horizontal coordinate representing –log_10_ (*P* value). Hypergeometric distribution; *P* < 0.01.

Next, we explored the heterogeneity and possible functions of myeloid cells and neutrophils in the process of carcinogenesis. To begin, we obtained 15,468 myeloid cells and reclustered them into 12 subsets. Two subsets of myeloid cells were annotated as monocytes (Mono-C1/-C2, *CD14^hi^CD68^lo^CSF1R^lo^*), and the other 5 subsets were classified as macrophages (Mac-C1/-C2/-C3/-C4/-C5, *CD68^hi^CSF1R^hi^*) (Figure 3, A and B, and Supplemental Table 8) (27). Each macrophage subset was distinguished by a specific marker (Mac-C1, *CCL3*^+^; Mac-C2, *CCL18*^+^; Mac-C3, *LYVE1*^+^; Mac-C4, *TREM2*^+^; Mac-C5, *SPP1*^+^). Apart from monocytes and macrophages, 5 subsets remained, including conventional DCs (cDCs) (28) (cDC-C1, *XCR1^+^*; cDC-C2, *CD1C^+^*; cDC-C3, *FCER1A^+^*; cDC-C4, *LAMP3^+^*) and plasmacytoid DCs (pDCs) (*LILRA4^+^*). Mac-C4 cells highly expressing *TREM2* showed a progressive increase from normal to OSCC tissues, and the percentages of activated cDCs (cDC-C4) and pDCs were significantly higher in OSCC tissues (*P* < 0.05, Supplemental Figure 3A and Supplemental Table 9). Analysis showed that almost all subpopulations of myeloid cells in the TME expressed the immune-inhibitory ligand *LGALS9* (encoding galectin 9) and *CD86* (Figure 3C), suggesting that these myeloid cells have strong T cell–suppressive functions. In addition, the interaction analysis revealed that myeloid cells in OSCC tissues showed extensive immune-inhibitory ligand-receptor binding with CD4^+^ and CD8^+^ T cells (Figure 3D). The expression levels of these immune-inhibitory ligands and the interaction intensities were lowest in normal tissues, higher in OLK tissues, and highest in OSCC tissues. These results suggest that the myeloid cells gradually formed an immune-inhibitory microenvironment during the process of oral carcinogenesis.

Next, 5,049 neutrophils were reclustered into 5 subgroups: Neutro-C1 (*IL1B^+^*), Neutro-C2 (*S100A12^+^*), Neutro-C3 (*ISG15^+^*), Neutro-C4 (*VEGFA^+^*), and Neutro-C5 (*CD274^+^*) (Figure 3, E and F, and Supplemental Table 10). The relative abundance of Neutro-C4 and -C5 showed a stepwise increase (Figure 3G and Supplemental Table 11). We also inferred the possible functions of Neutro-C4 and Neutro-C5. Neutro-C4 highly expressed *VEGFA* (the pro-hematopoietic factor), and their highly expressed genes were significantly enriched in the VEGFA/VEGFR2 pathway according to the Reactome database (*P* < 0.01, Supplemental Figure 3, B and C), suggesting that Neutro-C4 may promote angiogenesis. In addition, Neutro-C5 highly expressed *CD274*, and their highly expressed genes were enriched in the PD-1 signaling pathway (Figure 3H and Supplemental Figure 3D), suggesting that Neutro-C5 may also inhibit T cell function through the PD-L1/PD-1 signaling pathway. The highly expressed genes of Neutro-C1, -C2, and -C3 were not enriched in the VEGFA/VEGFR2 or PD-1 signaling pathways (Supplemental Figure 3E).

### A myofibroblast subtype expresses TDO2 in oral carcinogenesis.

**Figure 4:**
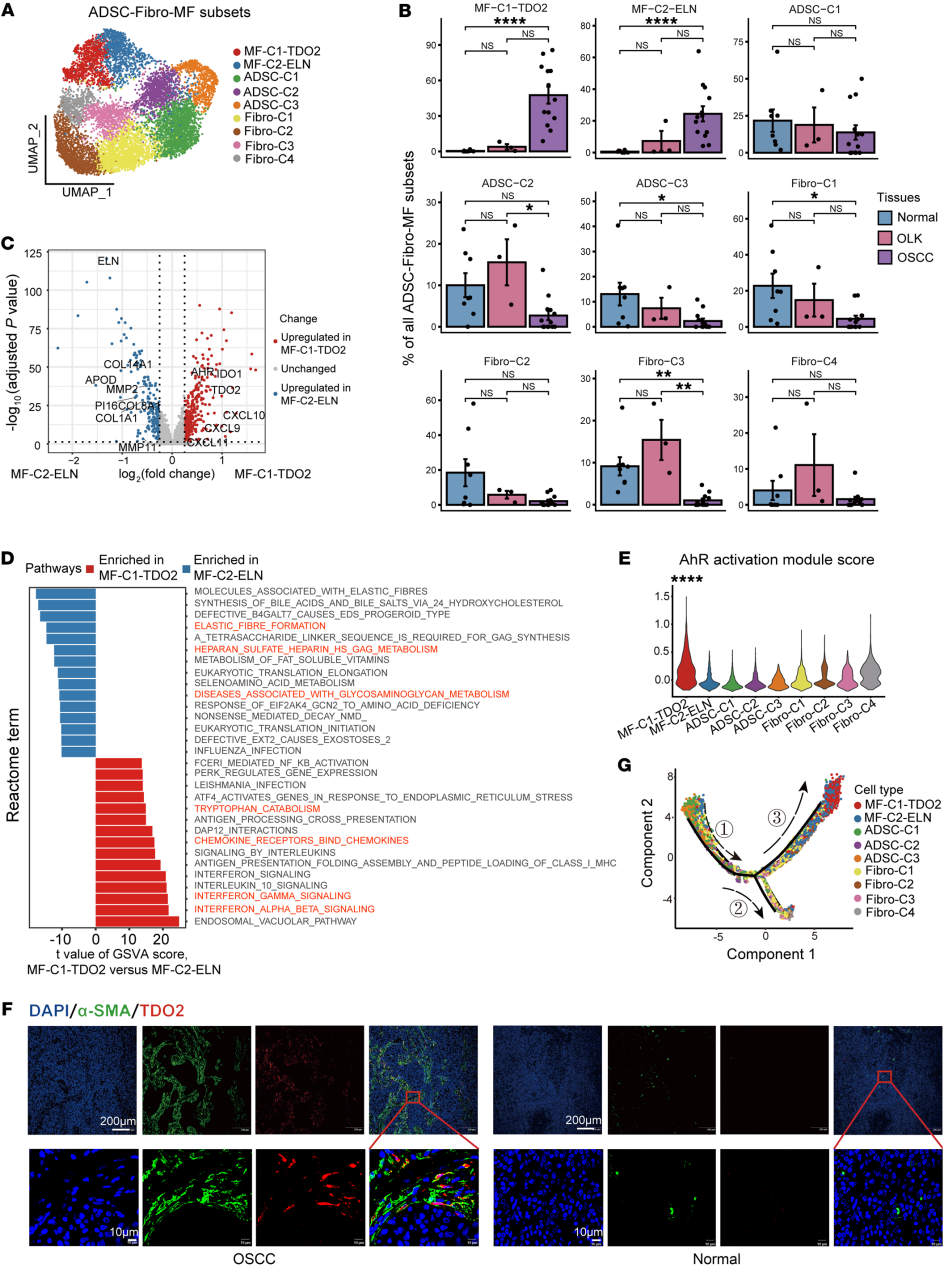
Subsets of ADSC-Fibro-MF cells and a subtype of myofibroblasts expressing *TDO2* in OSCC. (**A**) UMAP plot showing the distribution of ADSC-Fibro-MF subsets, with each color representing a cell subset. (**B**) Bar plots showing the percentage of ADSC-Fibro-MF subsets among total ADSC-Fibro-MF cells in adjacent normal, OLK, and OSCC tissues. *P < 0.05,**P < 0.01, and ****P < 0.0001, by Kruskal-Wallis test followed by Bonferroni’s multiple-comparison test. (**C**) Volcano plot showing differentially expressed genes between MF-C1-TDO2 and MF-C2-ELN. Red dots represent genes that were significantly upregulated in MF-C1-TDO2 [log_2_(fold change) >1, adjusted *P* < 0.05]; blue dots represent genes that were significantly upregulated in MF-C2-ELN [log_2_(fold change) <–1, adjusted *P* < 0.05]; and gray dots represent genes with no significant difference. (**D**) Bar plot showing the top 15 highest differential pathways between MF-C1-TDO2 and MF-C2-ELN. Red bars represent pathways that were enriched in MF-C1-TDO2, and blue bars represent pathways that were enriched in MF-C2-ELN. (**E**) Violin plot showing the score for the AhR activation module among ADSC-Fibro-MF subsets. ****P < 0.0001, by Wilcoxon rank-sum test. (**F**) IF staining images showing the expression intensity of α-SMA (green) and TDO2 (red) in OSCC and normal adjacent tissue. Images in the first row were obtained at ×10 magnification (scale bars: 200 μm). Images in the second row were obtained at ×40 magnification (scale bars: 10 μm). (**G**) UMAP plot showing the differentiation trajectory of ADSC-Fibro-MF cells and the distribution of each cell subset on the trajectory. Branch 1 is mainly composed of ADSCs, branch 2 is mainly composed of fibroblasts, and branch 3 is mainly composed of myofibroblasts. The numbers indicate the branches.

We next reclustered stromal cells into 6 major cell types (29–31): ADSCs (*CD34^+^NT5E^+^CFD^+^*), fibroblasts (*COL1A1^hi^THY1^hi^CRABP1^hi^ACTA2^lo^CD34^lo^*), myofibroblasts (*THY1^+^POSTN^+^ACTA2^+^*), smooth muscle cells (SMCs) (*ACTA2^+^MCAM^+^*), myoblasts (*MYF5^+^PAX7^+^*), and melanocytes (*MLANA^+^PMEL^+^*) (Supplemental Figure 4, A and B). The proportion of myofibroblasts gradually increased from normal to OLK to OSCC tissues and accounted for more than 50% of the stromal cells in OSCC tissues (Supplemental Figure 4C). Since previous studies reported mutual transformation among ADSCs, fibroblasts, and myofibroblasts (11), these 3 subgroups were selected for further analysis and classified into 9 subgroups: 3 subgroups of ADSCs (32, 33) (ADSC-C1, *VEGFD*^+^; ADSC-C2, *SFRP1*^+^; ADSC-C3, *MFAP5*^+^), 4 subgroups of fibroblasts (Fibro-C1, *CCL19*^+^; Fibro-C2, *IGF1*^+^; Fibro-C3, *THSD4*^+^; Fibro-C4, *IGFBP2*^+^), and 2 subgroups of myofibroblasts (MF-C1-TDO2, *TDO2*^+^; MF-C2-ELN, *ELN*^+^) (Figure 4A, Supplemental Figure 4D, and Supplemental Table 12). MF-C1-TDO2 and MF-C2-ELN myofibroblasts were nearly absent in normal tissues, slightly more common in OLK tissues, and substantially more abundant in OSCC tissues (Figure 4B and Supplemental Table 13).

To explore the potential roles of myofibroblasts in OSCC, we performed differential gene expression analysis between the MF-C1-TDO2 and MF-C2-ELN myofibroblast subpopulations (Supplemental Table 14). Genes upregulated in MF-C1-TDO2 included T cell chemokines such as *CXCL9/-10/-11* and the tryptophan (Trp) catabolic enzymes *TDO2* and *IDO1*, as well as their downstream target the aryl hydrocarbon receptor (AhR) (encoded by *AHR*) (Figure 4C). Genes upregulated in MF-C2-ELN included those related to matrix degradation and elastases, such as *MMP11*, *MMP2*, *ELN*, and *PI16*. Further gene set variation analysis (GSVA) of the transcriptional profiles of the 2 cell populations using the Reactome database revealed that MF-C1-TDO2 was mainly enriched in IFN-related signaling pathways (INTERFERON_GAMMA-_SIGNALING), the chemokine and chemokine receptor binding pathway (CHEMOKINE-_RECEPTORS_BIND_CHEMOKINES), and the Trp catabolic pathway (TRYPTOPHAN_CATABOLISM) (Figure 4D and Supplemental Table 15), suggesting that MF-C1-TDO2 myofibroblasts have a strong immune cell recruitment function and perform Trp catabolism. In contrast, GSVA of MF-C2-ELN primarily indicated enrichment in extracellular matrix metabolic pathways, including the elastin formation pathway (ELASTIC_FIBRE_FORMATION), the heparan sulfate metabolism pathway (HEPARAN_SULFATE_HEPARIN_HS_GAG_METABOLISM), and the glycosaminoglycan metabolism pathway (DISEASES_ASSOCIATED_WITH_GLYCOSAMINOGLYCAN_METABOLISM), suggesting that MF-C2-ELN myofibroblasts play a role in extracellular matrix remodeling in the TME. The AhR activation module score of MF-C1-TDO2 myofibroblasts was significantly higher than the scores for the other subpopulations (*P* < 0.0001, Figure 4E). These results suggest that MF-C1-TDO2 myofibroblasts may possess a strong chemotactic function and perform Trp catabolism. Trp catabolism has been found to influence the function of tumor-infiltrating T cells (34). Furthermore, MF-C1-TDO2 myofibroblasts were found in all OSCC tissues and were slightly enriched in OLK tissues, but they were nearly absent in normal tissues (Supplemental Figure 5A), suggesting that this subset of myofibroblasts is a typical hallmark of the process of oral carcinogenesis.

The above results suggest that MF-C1-TDO2 is a subpopulation of myofibroblasts expressing *TDO2*, but it is not clear whether *TDO2* is expressed in other cell subsets in the TME of OSCC. According to the UMAP plots, *TDO2* was exclusively expressed in a subset of stromal cells but not in immune cells or endothelial cells, and especially not in epithelial cells (Supplemental Figure 4E). These findings were confirmed by immunofluorescence (IF) imaging, which illustrated that TDO2 was predominantly expressed in α-SMA^+^ myofibroblasts in OSCC, whereas it was nearly absent in normal tissues (Figure 4F). These results suggest that *TDO2* is specifically expressed in MF-C1-TDO2 myofibroblasts in the TME of OSCC.

To further explore the developmental state of MF-C1-TDO2 myofibroblasts, a pseudotime trajectory was constructed via the R package monocle2 (Figure 4G and Supplemental Figure 4F). The pseudotime trajectory showed that ADSCs or fibroblasts were polarized into myofibroblasts during oral carcinogenesis. Notably, along branch 3, MF-C1-TDO2 myofibroblasts were located at the end; this differentiation process was accompanied by upregulation of *ACTA2*, *CXCL9*, *CXCL10*, and *TDO2* (Supplemental Figure 4G), suggesting that MF-C1-TDO2 myofibroblasts were the terminally differentiated. To summarize, MF-C1-TDO2 myofibroblasts were the terminally differentiated cells exclusively expressing *TDO2*, a Trp catabolic enzyme (TCE), indicating that MF-C1-TDO2 is a stable myofibroblast subset that participates in regulating T cell immune function in the TME of OSCC.

### MF-C1-TDO2 myofibroblasts attract T cells and shield tumor cells from T cell attacks.

**Figure 5:**
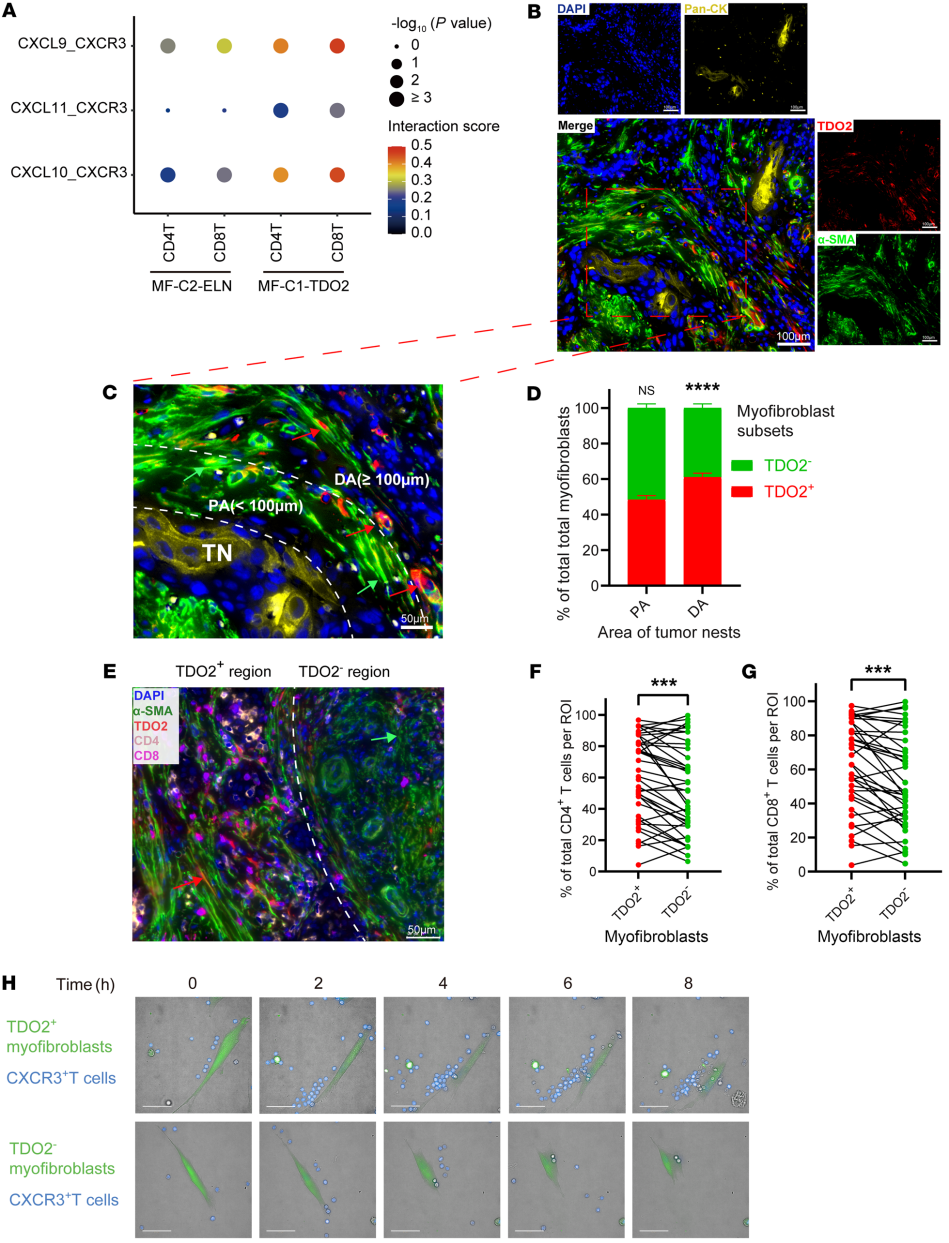
MF-C1-TDO2 myofibroblasts attract T cells and shield tumor cells from T cell attacks. (**A**) Dot plot showing the interaction intensity between chemokines (CXCL9/-10/-11) from myofibroblasts (MF-C2-ELN or MF-C1-TDO2) and the chemokine receptor CXCR3 on CD4^+^ and CD8^+^ T cells according to the CellPhoneDB analysis. (**B**) mIHC results showing the spatial localization of TDO2^+^ myofibroblasts (TDO2^+^α-SMA^+^), TDO2^–^ myofibroblasts (TDO2^–^α-SMA^+^), and cancer cells (Pan-CK^+^). Scale bars: 100 μm. (**C**) Representative image (Pt10_Ca) showing the spatial distribution of TDO2^+^ (red) and TDO2^–^ myofibroblasts (green) in the proximal area (PA) (PA <100 μm from the tumor nest [TN] border) and the distal area (DA) (DA ≥100 μm from the tumor nest border) of tumor nests. Scale bar: 50 μm. Green arrows indicate examples of TDO2^–^ cells in the proximal area; red arrows indicate examples of TDO2^+^ cells in the distal area. (**D**) Quantitative analysis of the proportions of TDO2^+^ and TDO2^–^ myofibroblasts in the PA and the DA of tumor nests. (**E**) Representative images (Pt10_Ca) showing the spatial distribution of CD4^+^ (orange) and CD8^+^ (purple) T cells around TDO2^+^ (radius <50 μm) and TDO2^–^ (radius <50 μm) myofibroblasts. Scale bar: 50 μm. Red arrow indicates an example of a TDO2^+^ cell; green arrow indicates an example of a TDO2^–^ cell. (**F** and **G**) Quantitative analyses of the proportions of (**F**) CD4^+^ and (**G**) CD8^+^ T cells around TDO2^+^ or TDO2^–^ myofibroblasts. ROI, region of interest. (**H**) High-content cell imaging showing the difference between the CXCR3^+^CD3^+^ T cell (blue) chemoattraction toward TDO2^+^ and TDO2^–^ myofibroblasts (green) from OSCC tissue at different time points in vitro. Scale bars: 50 μm. ***P < 0.001 and ****P < 0.0001, by 2-tailed Student’s *t* test (**D**, **F**, and **G**).

We further investigated the crosstalk between myofibroblasts and T cells in the TME. Analysis of the interactions between MF-C1-TDO2 myofibroblasts and CD8^+^ T or CD4^+^ T cells using the CellPhoneDB revealed that, in comparison with MF-C2-ELN myofibroblasts, MF-C1-TDO2 myofibroblasts in tumor tissue had stronger chemotaxis toward both CD8^+^ T cells and CD4^+^ T cells (Figure 5A). These results indicate that MF-C1-TDO2 myofibroblasts in the TME mainly attracted T cells through the binding of *CXCL9/-10/-11* and *CXCR3*. Similar to a previous description (35), multiplex immunohistochemical staining (mIHC) showed that in OSCC samples, myofibroblasts (α-SMA^+^) were mainly located on the periphery of tumor nests (Pan-CK^+^) (Figure 5B). And mIHC revealed that cells in OSCC tumor nests (Pan-CK^+^) were TDO2^–^, suggesting that OSCC tumor cells were negative for TDO2. In addition, 4 representative fields from the whole-slide scan images (*n* = 10) were captured, resulting in 40 fields in total for further quantitative analysis (Supplemental Figure 5C). The relative proportions of TDO2^+^ and TDO2^–^ myofibroblasts between the proximal area (<100 μm from the tumor nest border) and the distal area (≥100 μm from the tumor nest border) of tumor nests in each field from whole-slide scan images were calculated as previously described (Figure 5, C and D) (36). The results showed that TDO2^+^ myofibroblasts were enriched in the distal area, whereas TDO2^–^ myofibroblasts were mainly located in the proximal area of the tumor nests. In addition, quantitative analyses using StrataQuest software (TissueGnostics) demonstrated that the proportions of both CD4^+^ T cells and CD8^+^ T cells were more significantly enriched around TDO2^+^ myofibroblasts (radius <50 μm) in comparison with TDO2^–^ myofibroblasts (radius <50 μm) in each field (*P* < 0.001, Figure 5, E–G). These results suggest that TDO2^+^ and TDO2^–^ myofibroblasts differ in spatial distribution around tumor nests, in which TDO2^+^ myofibroblasts are located distally from tumor nests and possess a stronger capacity for chemotaxis toward CD4^+^ T cells and CD8^+^ T cells to shield tumor cells from T cell attacks in the TME.

These findings were verified by in vitro experiments. We sorted TDO2^+^ and TDO2^–^ myofibroblasts from tumor tissues to perform coculture experiments with CXCR3^+^CD3^+^ T cells on imaging plates. Since TDO2 is a cytoplasmic protein, as the surface marker, we used MCT4 (encoded by *SLC16A3*), which was exclusively coexpressed with *TDO2* in myofibroblasts according to the UMAP plots from the scRNA-Seq results (Supplemental Figure 6, A and B). These results were further confirmed by reverse transcription quantitative PCR (RT-qPCR) and IF staining (Supplemental Figure 6, C and D). Thus, TDO2^+^ and TDO2^–^ myofibroblasts were sorted by FACS on the basis of MCT4 expression. The expression levels of *CXCL9/-10/-11* were significantly higher in TDO2^+^ myofibroblasts than in their TDO2^–^ counterparts (Supplemental Figure 6E), which was consistent with the findings above (Figure 4C). High-content cell imaging demonstrated that, over time, TDO2^+^ myofibroblasts (green) had a much stronger T cell (blue) chemoattractant function compared with that of TDO2^–^ myofibroblasts (Figure 5H and Supplemental Videos 1 and 2). This result further confirms the strong T cell chemotactic function of TDO2^+^ myofibroblasts in the TME. To summarize, TDO2^+^ myofibroblasts were mainly located distally from tumor nests, and these cells attracted CD4^+^ T cells and CD8^+^ T cells through the CXCL9/CXCL10/CXCL11/CXCR3 axis, which may have prevented T cells from accessing tumor nests.

In addition, we analyzed the interplay between myofibroblasts and macrophages using the CellPhoneDB. We found that, compared with MF-C2-ELN myofibroblasts, MF-C1-TDO2 myofibroblasts in tumor tissue had a stronger ability for chemotaxis toward macrophages and enhanced interactions with the notch signaling pathway in macrophages (Supplemental Figure 6G). In addition, we found that there were interactions between macrophages and MF-C1-TDO2 myofibroblasts in the IL-1 pathway (Supplemental Figure 6H). A previous study reported that IL-1 can induce the proliferation of fibroblasts (37). Therefore, we speculate that macrophage-derived IL-1 may induce the proliferation of TDO2^+^ myofibroblasts in OSCC.

### TDO2^+^ myofibroblasts mediate T cell suppression.

**Figure 6:**
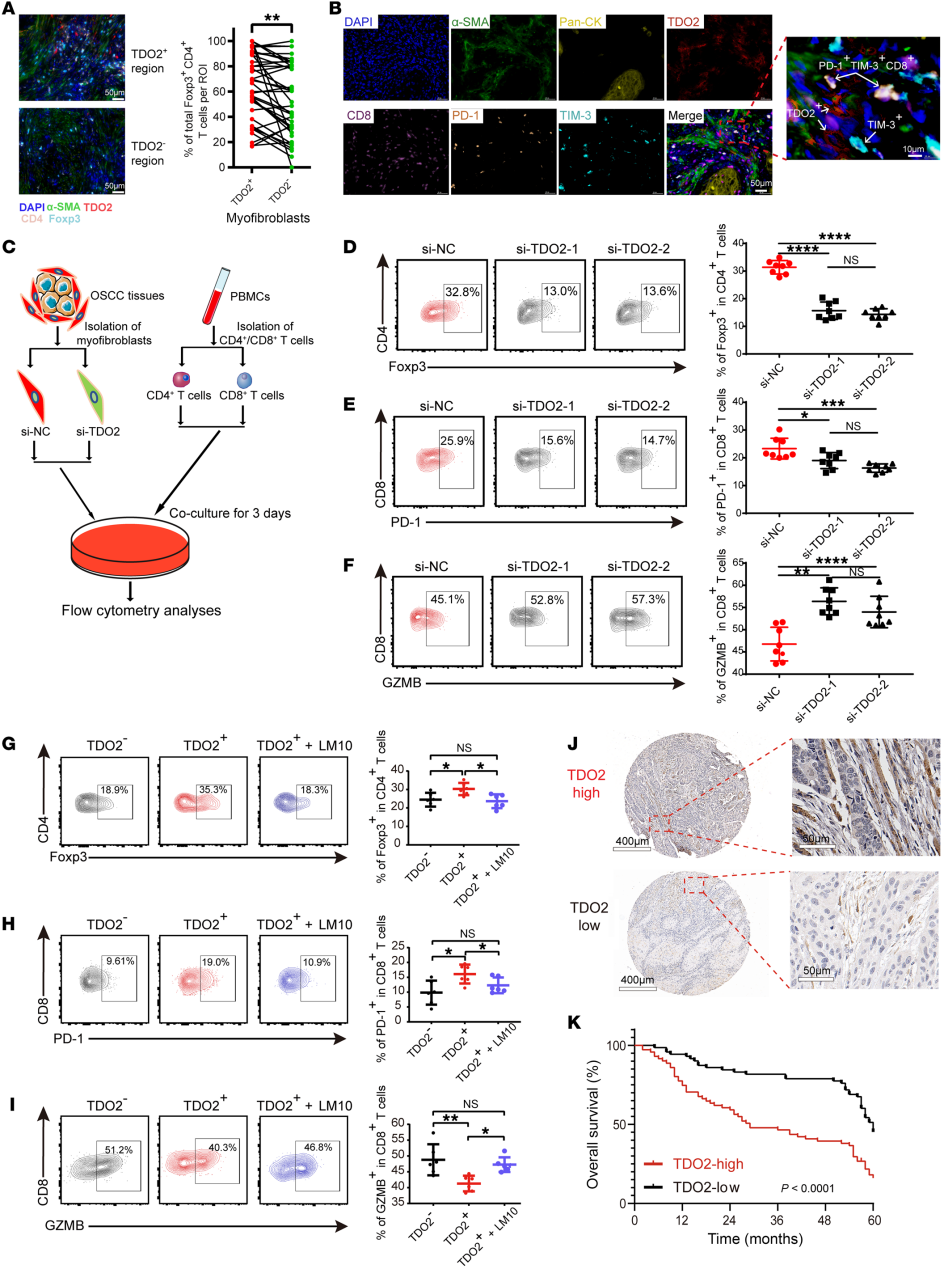
TDO2^+^ myofibroblasts mediate T cell suppression. (**A**) Representative images (Pt14_Ca) (left) and quantitative analyses (right) showing the spatial distribution and the proportions of Foxp3^+^CD4^+^ T cells (light blue) around TDO2^+^ (red) and TDO2^–^ (green) myofibroblasts (radius <50 μm). Scale bars: 50 μm. (**B**) Representative images (Pt14_Ca) showing the spatial distribution of PD-1^+^TIM-3^+^CD8^+^ T cells around TDO2^+^ myofibroblasts. Scale bars: 50 μm and 10 μm (enlarged inset). (**C**) Schematic diagram depicting the coculture strategy for control myofibroblasts (si-NC) and *TDO2*-knockdown myofibroblasts (si-TDO2) with CD4^+^ or CD8^+^ T cells. (**D**–**F**) Representative images of flow cytometry (left) and statistical results (right) showing the proportions of (**D**) Foxp3^+^CD4^+^ T cells, (**E**) PD-1^+^, and (**F**) GZMB^+^CD8^+^ T cells for the control group (si-NC) and the *TDO2*-knockdown groups (si-TDO2-1 and si-TDO2-2). (**G**–**I**) Representative flow cytometry (left) and statistical results (right) showing the proportion of (**G**) Foxp3^+^ T cells, (**H**) PD-1^+^, and (**I**) GZMB^+^CD8^+^ T cells after 3 days of coculturing with TDO2^–^ or TDO2^+^ myofibroblasts or with TDO2^+^ myofibroblasts plus the TDO2 inhibitor LM10. (**J**) Representative IHC images of TMAs. The H scores of representative TDO2^hi^ and TDO2^lo^ images were 141.9 and 68.0, respectively. Scale bars: 400 μm (left) and 50 μm (right). (**K**) OS curve between TDO2^hi^ (*n* = 71) and TDO2^lo^ (*n* = 71) cohorts of patients with OSCC according to the staining intensity of IHC images. *P* < 0.0001, by log-rank test. (**A** and **D**–**I**) *P < 0.05, **P < 0.01, ***P < 0.001, and ****P < 0.0001, by 1-way ANOVA followed by Bonferroni’s multiple-comparison test.

Next, we determined whether TDO2^+^ myofibroblasts exert an immunoregulatory effect on T cells after attracting them. A correlation analysis showed that the relative proportion of MF-C1-TDO2 myofibroblasts was positively correlated with the abundance of terminally exhausted CD8^+^ T cells (CD8-C5), transitory exhausted CD8^+^ T cells (CD8-C9), resting Tregs (CD4-C2), and activated Tregs (CD4-C5) (Supplemental Figure 5B). Furthermore, mIHC demonstrated that Foxp3^+^CD4^+^ T cells were significantly more abundant around TDO2^+^ myofibroblasts than TDO2^–^ myofibroblasts (*P* < 0.01, Figure 6A), and we also found that TDO2^+^ myofibroblasts and terminally exhausted CD8^+^ T cells (TIM-3^+^PD-1^+^CD8^+^ T cells) tended to be in close proximity to one another (Figure 6B). As previously reported, TDO2 expressed on tumors is a TCE that degrades Trp into kynurenine (Kyn), which inhibits the function of CD4^+^ and CD8^+^ T cells (38). We hypothesized that TDO2^+^ myofibroblasts might also mediate T cell suppression. To test this hypothesis, we transfected OSCC-derived myofibroblasts with an siRNA to knock down *TDO2* expression in vitro. The coculture experiment between myofibroblasts and CD4^+^ or CD8^+^ T cells isolated from PBMCs is shown in Figure 6C. T cells were collected and analyzed by flow cytometry after 3 days according to the gating strategy shown in Supplemental Figure 6F. The coculture results showed that silencing of *TDO2* in myofibroblasts resulted in downregulated expression of Foxp3 in CD4^+^ T cells and of PD-1 in CD8^+^ T cells, whereas the secretion of granzyme B (GZMB) increased in CD8^+^ T cells (Figure 6, D–F), suggesting that OSCC-derived myofibroblasts inhibited the effector functions of CD4^+^ or CD8^+^ T cells via *TDO2* and that silencing of *TDO2* in myofibroblasts could effectively reverse the suppressive status of both CD4^+^ and CD8^+^ T cells.

In addition, TDO2^+^ and TDO2^–^ myofibroblasts were isolated from OSCC tissues by FACS and cocultured with CD4^+^ or CD8^+^ T cells isolated from PBMCs. The proportion of Foxp3^+^ Tregs was significantly increased in the TDO2^+^ group in comparison with the TDO2^–^ group (*P* < 0.05, Figure 6G). Furthermore, we also observed that TDO2^+^ myofibroblasts were associated with upregulated PD-1 expression on CD8^+^ T cells (*P* < 0.05, Figure 6H), and the effector function of CD8^+^ T cells was inhibited, as reflected by reduced production of GZMB (*P* < 0.01, Figure 6I). All of these results suggest that TDO2^+^ myofibroblasts might mediate T cell suppression, and the suppressive status of T cells was reversed by the addition of the TDO2 inhibitor LM10 (39).

Since TDO2 was upregulated in myofibroblasts in OSCC tissues and mediated T cell inhibition in the TME, we next investigated the prognostic value of TDO2^+^ myofibroblasts in patients with OSCC. The tissue microarrays (TMAs) of an independent cohort of patients with OSCC were stained with TDO2 and quantified with H scores (median = 86.5; range, 0.8–195.2). Consistent with the description above, TDO2 was almost located in the stromal area around cancer nests. The patients (*n* = 142) were dichotomized into TDO2^hi^ (H scores ≥86.5; *n* = 71) and TDO2^lo^ (H scores <86.5; *n* = 71) groups according to the median H score (Figure 6J). Representative images of TDO2^hi^ and TDO2^lo^ are shown in Figure 6J. Kaplan-Meier curves showed that the 5-year OS of the TDO2^hi^ group was significantly worse than that of the TDO2^lo^ group (*P* < 0.0001, Figure 6K). We performed Cox regression analysis of 142 patients with OSCC in TMAs, among which the variables included sex, age, smoking status, alcohol use, differentiation status, tumor site, tumor (T) stage, node (N) stage, radiotherapy, chemotherapy, and TDO2 status. The results showed that, for univariate and multivariate analyses, the N stage and TDO2 status were the risk factors with significant differences (*P* < 0.05, Table 1). These survival analyses revealed that TDO2^+^ myofibroblasts are associated with a worse prognosis for patients with OSCC .

### Inhibition of TDO2 prevented the progression of malignant transformation in oral carcinogenesis in murine models.

**Figure 7:**
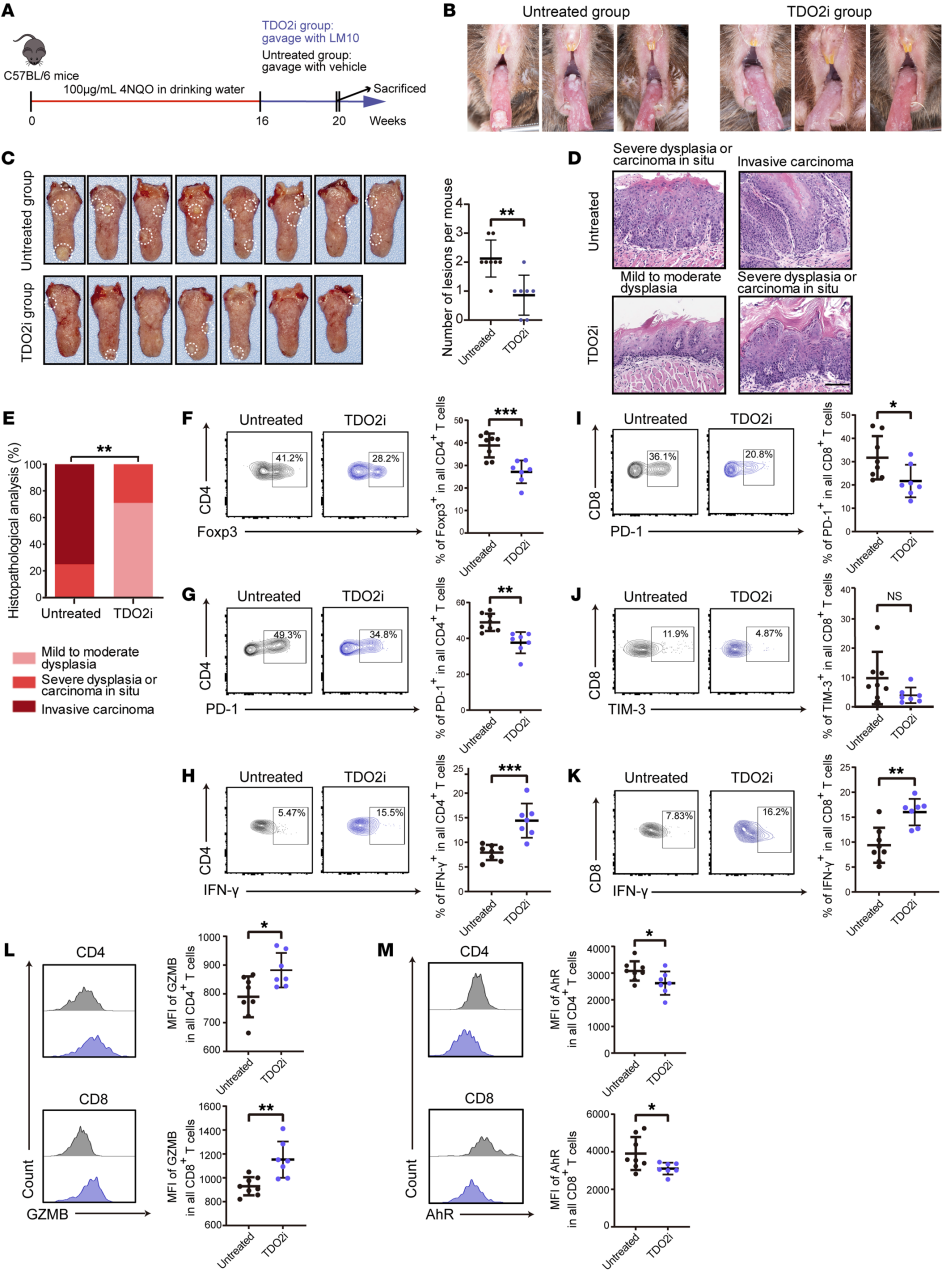
Inhibition of TDO2 prevents the formation of OSCC in 4NQO-induced carcinogenic murine models. (**A**) Schematic plot showing the induction of OSCC by 4NQO in C57BL/6 mice and the administration strategies for the TDO2i group (*n* = 7) and the untreated group (*n* = 8). (**B**) Six representative intraoral lesions on the tongues of mice in the TDO2i and untreated groups. (**C**) Macroscopic lesions on the tongues (left) and statistical results (right) for mice in the TDO2i and untreated groups. The dotted circles indicate macroscopic cauliflower-like lesions. (**D**) Representative microscopic images of the TDO2i and untreated groups following H&E staining. Scale bar: 100 μm. (**E**) Comparison of the tongue lesions (mild-to-moderate dysplasia, severe dysplasia or carcinoma in situ, and invasive carcinoma) in mice from the TDO2i and untreated groups. **P < 0.01, by Fisher’s exact test. (**F**–**H**) Representative flow cytometry (left) and statistical results (right) showing the proportions of (**F**) Foxp3^+^, (**G**) PD-1^+^ and (**H**) IFN-γ^+^CD4^+^ T cells for the TDO2i and untreated groups. (**I**–**K**) Representative flow cytometry (left) and statistical results (right) showing the proportions of (**I**) PD-1^+^, (**J**) TIM-3^+^, and (**K**) IFN-γ^+^CD8^+^ T cells for the TDO2i and untreated groups. (**L** and **M**) Representative flow cytometry (left) and statistical results (right) showing the median fluorescence intensity (MFI) of (**L**) GZMB and (**M**) AhR for CD4^+^ (upper) and CD8^+^ (lower) T cells from the TDO2i and untreated groups. *P < 0.05, **P < 0.01, and ***P < 0.001, by 2-tailed Student’s *t* test (**C** and **E**–**M**).

To examine the effects of TDO2 inhibitors on OSCC in vivo, we established a 4NQO-induced carcinogenesis model in immunocompetent mice. Briefly, C57BL/6 mice (*n* = 15) were divided into a TDO2 inhibitor–treated (TDO2i) group (*n* = 7) and an untreated group (*n* = 8), and mice in both groups were given drinking water containing 100 μg/mL 4-nitroquinoline-1 oxide (4NQO) once per week. After 16 consecutive weeks of induction, when oral precancerous lesions had formed on the murine oral tongue mucosa, mice in the TDO2i group were administered the TDO2 inhibitor LM10 every day by oral gavage, whereas the untreated group received the same volume of vehicle (Figure 7A). By week 20, we observed that the mice in the TDO2i group showed fewer macroscopic cauliflower-like lesions on the tongue than did mice in the untreated group (Figure 7, B and C). Microscopic observation of H&E staining (Figure 7D) showed that the TDO2i group had no invasive carcinoma lesions (0 of 7) but more cases of mild-to-moderate dysplasia (early lesions, 5 of 7, 71%) on their tongues, whereas 6 cases of invasive carcinoma (6 of 8, 75%), 2 cases of preinvasive carcinoma (severe dysplasia or carcinoma in situ, 2 of 8, 25%), and no cases of mild-to-moderate dysplasia were noted in the untreated group (Figure 7E), indicating that administration of the TDO2 inhibitor significantly suppressed the formation of OSCC in 4NQO-treated mice (*P* < 0.01). Flow cytometric analysis showed that the TDO2i group had significantly fewer Tregs (Foxp3^+^ CD4^+^) in lesions than did the untreated group (*P* < 0.001, Figure 7F). Downregulation of PD-1 on CD4^+^ T cells and increased production of IFN-γ showed that the effector functions of CD4^+^ T cells were enhanced by TDO2 inhibition (Figure 7, G and H). In addition, downregulation of PD-1 and T cell Ig and mucin domain 3 (TIM-3) indicated that the exhausted state of intralesional CD8^+^ T cells was partially reversed (Figure 7, I and J), and increased production of IFN-γ and GZMB showed that the cytotoxic functions of intralesional CD8^+^ T cells were also strengthened in comparison with the untreated group (Figure 7, K and L). Furthermore, AhR was downregulated in CD4^+^ and CD8^+^ T cells from the TDO2i group (Figure 7M), indicating that the recovery of T cell functions was dependent on the blockade of the TDO2/AhR axis. Similar results were also obtained by draining lymph node (dLN) analysis of murine OSCC (Supplemental Figure 7), indicating that the T cell functions in the dLN might also be inhibited by TDO2^+^ myofibroblasts. Taken together, these results reveal that targeting TDO2^+^ myofibroblasts successfully enhanced the effector functions of T cells while attenuating their inhibitory states, preventing the progression of OSCC in murine models.

**Figure 8:**
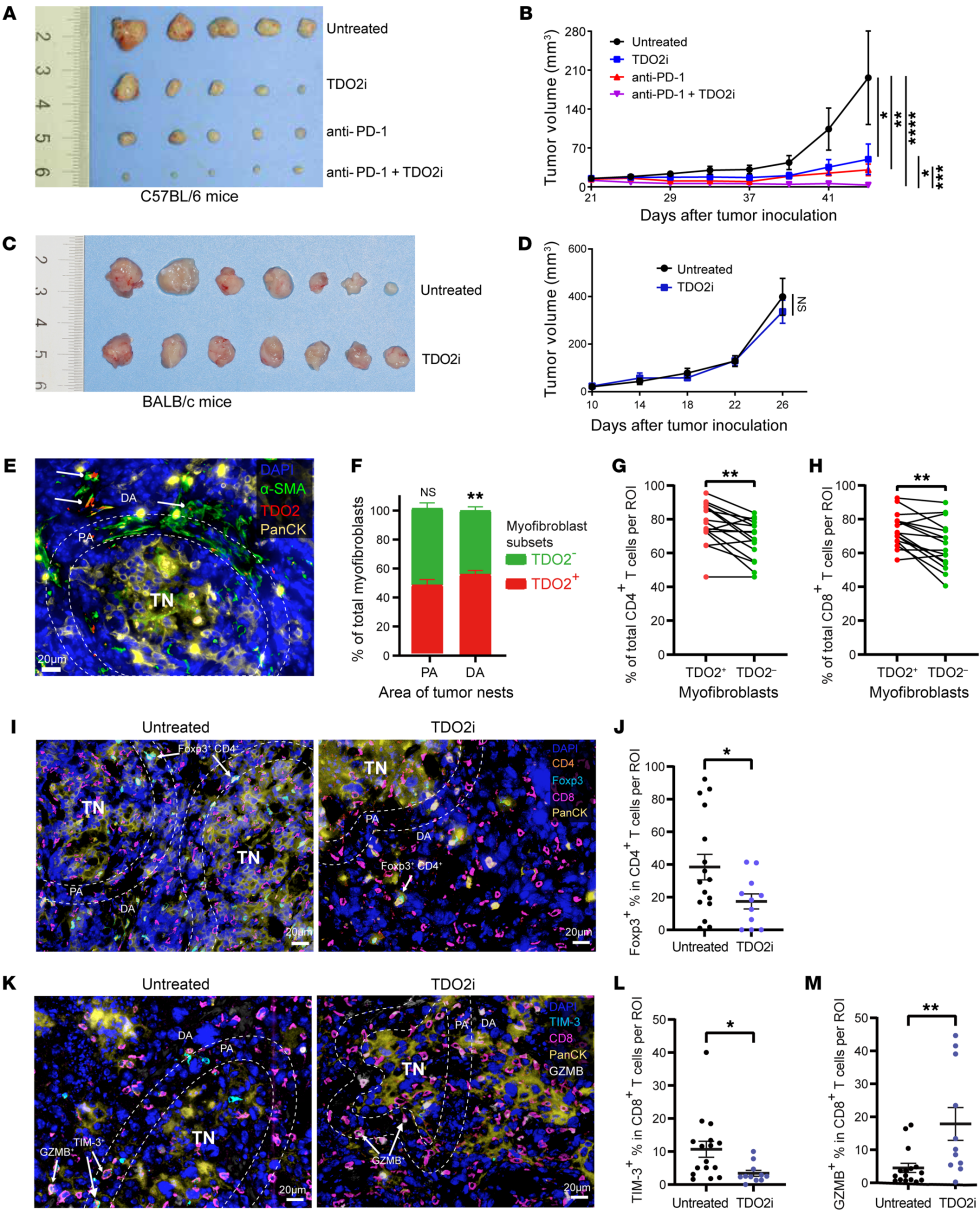
TDO2 inhibition enhances the effector function of CD8^+^ T cells to exert antitumor immunity. (**A** and **B**) Gross appearance of the tumor mass (**A**) and kinetics of the tumor volume (mm^3^) (**B**) were measured and documented for C57BL/6 mice in the untreated group, the anti–PD-1 group, the TDO2i group, and the anti–PD-1 plus TDO2i group. (**C** and **D**) Gross appearance of the tumor mass (**C**) and kinetics of the tumor volume (mm^3^) (**D**) were measured and documented for BALB/c nude mice in the untreated group and the TDO2i group. (**E**) Representative image showing the spatial distribution of TDO2^+^ or TDO2^–^ myofibroblasts in the PA and DA. Scale bar: 20 μm. (**F**) Quantitative analysis of the proportions of TDO2^+^ and TDO2^–^ myofibroblasts in the PA and DA. (**G** and **H**) Quantitative analyses of the proportions of (**G**) CD4^+^ and (**H**) CD8^+^ T cells around TDO2^+^ or TDO2^–^ myofibroblasts from murine tumor tissues. (**I** and **J**) Representative images (**I**) and quantitative analyses (**J**) showing the spatial distribution and proportions of Foxp3^+^ CD4^+^ T cells located in the PA of tumor tissues from mice in the untreated and TDO2i groups. Scale bars: 20 μm. (**K**–**M**) Representative images (**K**) and quantitative analyses showing the spatial distribution and proportions of (**L**) TIM-3^+^CD8^+^ T cells and (**M**) GZMB^+^CD8^+^ T cells located in the PA of tumor tissues from mice in the untreated and TDO2i groups. Scale bars: 20 μm. *P < 0.05, **P < 0.01, ***P < 0.001, and ****P < 0.0001, by repeated-measures analysis of means (**B** and **D**) and *P < 0.05 and **P < 0.01, by 2-tailed Student’s *t* test (**F**–**H** and **J**, **L**, and **M**). PA, less than 20 μm from the murine tumor nest border. DA, 20 μm or more from the murine tumor nest border. TN, tumor nest.

To further investigate whether TDO2 inhibitors can inhibit tumor growth and promote anti–PD-1 efficacy, we constructed a subcutaneous tumorigenesis model in C57BL/6 mice inoculated with murine OSCC cells (4MOSC2). We found that, compared with the untreated group, mice in the anti–PD-1 or TDO2i group showed reduced tumor volumes, and the combination of TDO2i plus anti–PD-1 resulted in the greatest tumor reduction (Figure 8, A and B), suggesting that the combination therapy could effectively inhibit tumor progression. In addition, we conducted in vivo experiments on BALB/c nude mice that lacked T cells in peripheral tissues. The nude mice were randomly divided into 2 groups and subcutaneously inoculated with murine OSCC cells (4MOSC2). Then, mice in the TDO2i group were administrated the TDO2 inhibitor LM10 by oral gavage, while mice in the untreated group were fed sterile water. As shown in Figure 8, C and D, there were no significant differences between the TDO2i and untreated groups. These results indicate that TDO2 inhibitors could inhibit tumor growth and promote anti–PD-1 efficacy and that the antitumor activity was dependent on T cells.

In addition, to test whether our results could be validated in animal models, we performed mIHC staining and statistical analyses of tumors from C57BL/6 mice. As the tumor nests in murine tumors were relatively smaller than those in humans, a shorter radius (20 μm) around the tumor nests was chosen to perform the statistical analyses. As shown in Figure 8, E–H, the murine tumors showed that TDO2^+^ myofibroblasts were also mainly located in the distal area of the tumor nests and that CD4^+^ and CD8^+^ T cells were mainly located around TDO2^+^ myofibroblasts, consistent with the results observed in human OSCC tissues (Figure 5, B–G). Next, we performed mIHC staining on murine tumor slides to evaluate T cell function in both the untreated group and the TDO2i group. We found that after TDO2 inhibition, in the proximal region from the tumor nests (<20 μm), the proportions of Tregs (Foxp3^+^CD4^+^) in CD4^+^ T cells and exhausted CD8^+^ T cells (TIM-3^+^CD8^+^) in CD8^+^ T cells were significantly reduced (*P* < 0.05, Figure 8, I–L). In addition, the percentages of effector T cells (GZMB^+^CD8^+^) in CD8^+^ T cells surrounding the tumor nests were significantly enriched (*P* < 0.01, Figure 8M), indicating that TDO2 inhibition enhanced the effector function of CD8^+^ T cells to exert the antitumor effect.

## Discussion

In the present study, we systematically profiled the dynamic changes in the microenvironment of OSCC and OLK by single-cell analysis. We found that CD4^+^ T cells differentiated into activated Tregs with highly immunosuppressive functions and highly exhausted CD4^+^ T cells and that CD8^+^ T cells consistently converted to an exhausted state. Further analysis showed that a subset of myofibroblasts was the key factor contributing to the immunosuppressive microenvironment of OSCC.

Previous studies have demonstrated that HNSCC, including OSCC, is a typical type of “inflamed” tumor that is characterized by high intratumoral T cell infiltration (40, 41), and large numbers of T cells have been found in samples from oral premalignant tissues (42, 43). Our results, consistent with these studies, revealed that malignant transformation of the oral mucosa was accompanied by an increased abundance of infiltrating T cells. However, these infiltrating T cells could not prevent the initiation and progression of OSCC, indicating that T cells in the immune microenvironment were functionally impaired. Previous scRNA-Seq analyses of HNSCC have focused on the partial epithelial-mesenchymal transition (pEMT) program (21) or the differences in the transcriptional landscape of immune cells between HNSCC and normal tissues (22), but the dynamic changes among different cell types during oral carcinogenesis remain less explored. In the present study, through RNA velocity analysis, we systematically inferred the dynamic changes undergone by CD4^+^ and CD8^+^ T cells during the process of oral carcinogenesis and found that both CD4^+^ and CD8^+^ T cells underwent a significant cell fate transition during this process. In brief, the majority of CD4^+^ T cells differentiated into immunosuppressive Tregs, whereas a minority of these cells differentiated into exhausted CD4^+^ T cells, and CD8^+^ T cells continuously differentiated from preexhausted into terminally exhausted CD8^+^ T cells. A previous study showed that CD8^+^ T cells were activated in the premalignant stage of liver cancer, but they rapidly became dysfunctional (44). All of these findings suggest that T cells become functionally impaired early in the premalignant stage of carcinogenesis, after which they transform into an inhibited phenotype.

Current efforts to improve the efficacy of cancer immunotherapy are mainly focused on reversing T cell exhaustion by exploiting pathways such as the PD-L1/PD-1 signaling pathway and the CD80/-86/CTLA4 pathway (5). However, the types of cells in the TME that drive the functional inhibition of T cells have not been comprehensively identified. Recent studies have shown that *TREM2*^+^ macrophages are the main subsets of tumor-associated macrophages (TAMs) that mediate immune suppression and resistance to immunotherapy (45, 46), confirming that targeting immune checkpoint molecules on T cells is not the only way to attenuate T cell dysfunction. Through cell-cell interaction analysis, we found that nearly all subsets of myeloid cells were capable of inhibiting T cell functions in the TME to some extent. Furthermore, we also noticed that 2 different subsets of tumor-associated neutrophils (TANs) were enriched in OSCC tissues; 1 subset induced angiogenesis, while the other inhibited T cells. Further studies are required to uncover the contributions and mechanisms of TANs in the progression of OSCC.

As the major subtype of stromal cells in the TME, whether myofibroblasts mediate T cell suppression and how they might accomplish this suppression remain largely unknown. Mechanistically, myofibroblasts inhibit CD8^+^ T cell infiltration (47), convert CD4^+^ T cells into Tregs in a TGF-β signaling–dependent manner (17, 48), and inhibit CD8^+^ T cells via the PD-L1/PD-L2/PD-1 pathway (49). Furthermore, myofibroblasts sequester CD8^+^ T cells to prevent them from attacking tumor cells via the CXCL12/CXCR4 signaling pathway (50). In the present study, we discovered that α-SMA^+^ myofibroblasts were the most predominant type of fibroblasts in the OSCC TME, and they were divided into 2 transcriptionally and functionally distinct subsets by scRNA-Seq: the ELN^+^ subset functions in matrix remodeling, whereas the TDO2^+^ subset functions in immune regulation. We found that TDO2^+^ myofibroblasts expressed high levels of CXCL9/-10/-11 and attracted both CD4^+^ and CD8^+^ T cells mainly through the CXCL9/CXCL10/CXCL11/CXCR3 axis, which seemed to contribute to the “inflamed” TME of OSCC, but further analysis revealed that TDO2^+^ myofibroblasts mainly resided in the distal area of tumor nests, and the T cells surrounding TDO2^+^ myofibroblasts had inhibitory phenotypes and rarely infiltrated into tumor nests.

Elyada et al. previously demonstrated that there are 3 distinct cancer-associated fibroblast (CAF) subtypes in pancreatic ductal adenocarcinoma, including myofibroblastic CAFs, inflammatory CAFs, and antigen-presenting CAFs (51). In the present study, we found that TDO2^+^ and ELN^+^ myofibroblasts were highly similar to myofibroblastic CAFs, while inflammatory CAF marker genes and antigen-presenting CAF marker genes were highly expressed in the subtypes of normal fibroblasts and ADSCs in OSCC. In addition, a previous study reported that there were 2 distinct myofibroblastic (α-SMA^+^) CAF subtypes in breast cancer: (a) FAP^–^α-SMA^+^ myofibroblasts, which mainly contribute to the regulation of the cytoskeleton, and (b) FAP^+^α-SMA^+^ myofibroblasts, which possess the ability to attract and retain Tregs (18). In the present study, we found that TDO2^+^ and ELN^+^ myofibroblasts both expressed *FAP*, which promoted immunosuppression by CAFs (52–54), indicating that TDO2^+^ and ELN^+^ myofibroblasts are immunosuppressive and associated with resistance to immunotherapy. Notably, TDO2^+^ myofibroblasts may have a stronger immunosuppressive function due to the expression of *TDO2,* which mediates T cell inhibition. These observations, resembling those of previous studies (50), indicate that TDO2^+^ myofibroblasts may form a “barrier” to prevent T cells from attacking tumors through chemokine and chemokine receptor binding while also causing T cell inhibition. However, the detailed mechanisms through which TDO2^+^ myofibroblasts mediate T cell suppression remain to be explored.

In addition to direct ligation of immune-inhibitory ligand-receptor pairs, amino acid metabolism has emerged as a key mechanism mediating immune suppression in the TME (55). TDO2 is a newly discovered TCE that degrades Trp into Kyn (38). Kyn enters cells and activates AhR, resulting in tumor proliferation and immune suppression. Unlike IDO1 and IDO2, which are widely expressed on various types of cells, including tumor cells, TAMs and DCs, TDO2 was found to be selectively expressed on liver cancer cells and brain tumor cells (56), and it was also found to have greater Kyn production ability in comparison with IDO1/-2 (38). In contrast, in the present study, through scRNA-Seq, we found that *TDO2* was specifically and highly expressed in a subgroup of myofibroblasts (TDO2^+^ myofibroblasts) in OSCC, whereas *TDO2* expression was absent on tumor cells according to scRNA-Seq analysis and mIHC staining. Although this subset of myofibroblasts can recruit CD8^+^ and CD4^+^ T cells via CXCL9/-10/-11, it can also induce CD4^+^ T cells to transform into Tregs and induce CD8^+^ T cell dysfunction through the TDO2 signaling pathway. Finally, our results showed that the silencing of *TDO2* in myofibroblasts and the TDO2 inhibitor LM10 reversed the transformation of Tregs and exhausted CD8^+^ T cells (39) and successfully enhanced the effector functions of T cells and attenuated their inhibitory states, preventing the progression of OSCC in murine models. Therefore, we believe that TDO2^+^ myofibroblasts play a key role in modulating immune responses in OSCC and are thus a promising target for immunotherapy in patients with OSCC.

## Methods

### Clinical samples.

For scRNA-Seq, fresh tissues from 13 OSCC samples, 3 OLK samples, and 8 adjacent normal tissues were obtained during surgical resection at the Hospital of Stomatology, Sun Yat-sen University (Guangzhou, Guangdong, China). Ten OSCC samples among these samples were chosen for further mIHC analysis. Five fresh tumor tissues from patients with OSCC were used to isolate the primary myofibroblasts for high-content real-time imaging, IF, RT-qPCR, and coculture systems. For IHC analyses, the expression of TDO2 was assessed using TMAs containing samples from 142 patients with primary OSCC who were treated at the Hospital of Stomatology, Sun Yat-sen University between January 2008 and December 2009. All specimens were confirmed histologically by H&E staining. The follow-up interval was calculated from the date of surgery to the date of death or last follow-up. The clinicopathological characteristics of the 142 patients with OSCC in TMAs are described in Supplemental Table 16.

### Mice.

Six-week-old male C57BL/6 and BALB/c nude mice were purchased from GemPharmatech.

### 4NQO-induced oral tumorigenesis.

Fifteen mice were divided into the untreated group (*n* = 8) and TDO2i group (*n* = 7). All mice were given sterile water containing 100 μg/mL 4NQO (MilliporeSigma) for 16 consecutive weeks. At week 16, the 4NQO was replaced with sterile water. For mice in the TDO2i group, the TDO2 inhibitor LM10 (CsnPharm) was dissolved with 2% DMSO in sterile water and administered at a dose of 160 mg/kg per day by oral gavage, while the mice in the untreated group were fed the same concentration of DMSO (2%) in water from week 16. The experiment lasted until the end of week 20 and was performed in a specific pathogen–free environment. Mice were sacrificed at week 20, and the dLNs of mice were harvested for flow cytometry, while the tongue lesions were divided into 2 parts: 1 for histopathological analysis and the other for flow cytometry.

### Inoculated murine tumor models.

The 4MOSC2 murine OSCC cell lines were used in the tumor model (57). Cells were cultured in keratinocyte media, passaged a maximum of 4 times, and tested for mycoplasma contamination before the in vivo experiments. 4MOSC2 tumor cells (*n* = 500,000) were subcutaneously inoculated into the right flank of C57BL/6 mice (*n* = 5 per group) or BALB/c nude mice (*n* = 7 per group). Mice in the TDO2i or combination group were administrated the TDO2 inhibitor LM10 by oral gavage, as described above, whereas mice in the untreated group were given sterile water. C57BL/6 mice in the anti–PD-1 or combination group were injected peritoneally with anti–PD-1 antibodies (8 mg/kg) starting from day 21 and every 3 days throughout the entire experiment, and mice in the untreated group were injected peritoneally with control IgG (8 mg/kg) with the same volume at the same time. An anti–mouse PD-1 antibody was purified from hybridoma (clone G4) culture supernatant (provided by Lieping Chen, Yale University, New Haven, Connecticut, USA). All tumors were measured with calipers every 4 days. The tumor volume was estimated using the following formula: (length × width^2^)/2. The tumors were dissected immediately after the mice were euthanized.

### Tissue dissociation and single-cell suspensions.

Fresh samples were trimmed, washed with Dulbecco’s PBS (D-PBS) (Thermo Fisher Scientific), minced, and dissociated using a Human Tumor Dissociation Kit (Miltenyi Biotec) according to the manufacturer’s guidelines (see Supplemental Methods).

### Preparation of scRNA-Seq libraries and sequencing.

Single-cell transcriptome sequencing was performed using the droplet-based 10X Genomics platform. Preparation of scRNA-Seq libraries and analysis of scRNA-Seq are described in the Supplemental Methods.

### H&E staining, IHC, IF, and mIHC.

The staining and analysis results of the H&E, IHC, IF, and mIHC experiments were checked by 2 certified pathologists. Details on the methods are described in the Supplemental Methods.

### Isolation and culturing of myofibroblasts from OSCC.

To isolate primary myofibroblasts from OSCC, OSCC tissues were immersed in PBS with an antibiotic and an antimycotic for 10 minutes. The isolation and culturing processes were performed according to previously described protocols (58) (see also Supplemental Methods).

### Gene knockdown by siRNA transfection.

siRNA-mediated gene knockdown of TDO2 was performed using Lipofectamine 2000 (Invitrogen, Thermo Fisher Scientific). The sequences of 2 siRNAs specific for TDO2 are listed in Supplemental Table 17. A scrambled nontargeting siRNA was used as a negative control (si-NC). The human OSCC myofibroblasts were seeded in 6-well culture plates (50,000 cells per plate) overnight and transfected with corresponding 50 nM siRNAs for 6 hours according to the manufacturer’s protocol.

### High-content, real-time imaging.

Myofibroblasts isolated and cultured from OSCC samples were stained with Ghost Dye Red 780 (13-0865-T100, Tonbo Biosciences) in darkness for 15 minutes, followed by incubation with rabbit anti-MCT4 primary antibodies (sc-376140, Santa Cruz Biotechnology) and subsequent incubation with Goat Anti–Mouse IgG H&L (DyLight 488) for 30 minutes in darkness at 4°C. Next, the myofibroblasts were sorted into MCT4^+^ (TDO2^+^) or MCT4^–^ (TDO2^–^) myofibroblasts via FACS according to the expression level of MCT4. Live CXCR3^+^CD3^+^ T cells were sorted from PBMCs from 6 healthy donors. Briefly, the sorted TDO2^+^ and TDO2^–^ myofibroblasts were added (3,000 cells per well) to 96-well microplates (CellCarrier-96 Ultra, PerkinElmer) and cultured for 6 hours for total adherence, followed by incubation with CFSE for 15 minutes at 37°C. Next, the myofibroblasts were washed and added to CXCR3^+^CD3^+^ T cells (10,000 cells per microplate). The microplates were then placed into a high-content imaging analysis system (Operetta CLS, PerkinElmer), and microscopic images of the cells were captured every 30 minutes for 8 hours.

### Coculturing of myofibroblasts and CD4^+^ and CD8^+^ T cells.

Myofibroblasts in the si-TDO2-1, si-TDO2-2, and si-NC groups were then seeded in 96-well plates (10,000 cells per well, 8 wells per group) for 6 hours for total adherence, respectively. For the in vitro TDO2 inhibitor study, the sorted TDO2^+^ and TDO2^–^ myofibroblasts were added to 96-well plates (10,000 cells per well) for 6 hours for total adherence. The myofibroblasts were divided into 3 groups (6 wells per group). TDO2^+^ myofibroblasts were added to the TDO2^+^ and TDO2^+^ LM10 group, while TDO2^–^ myofibroblasts were added to the TDO2^–^ group. The TDO2^+^ LM10 group was treated with the TDO2 inhibitor LM10 (5 μM per plate) (39), whereas the other 2 groups were treated with an equal volume of solvent. CD3^+^ T cells from PBMCs from 6 healthy donors were isolated by negative selection using magnetic cell separation (MACS) (human CD3^+^ T cell Isolation Kit, Miltenyi Biotec). CD4^+^ and CD8^+^ T cells were isolated from CD3^+^ T cells by positive selection using MACS (human CD4^+^ or CD8^+^ T cell Isolation Kit, Miltenyi Biotec). The isolated CD4^+^ and CD8^+^ T cells were incubated with CFSE for 15 minutes at 37°C before being added to 96-well plates (100,000 T cells per well). The coculture system was then incubated with anti-CD3 and anti-CD28 antibodies (250 ng/mL) and IL-2 (10 ng/mL). Cells were harvested for flow cytometric analyses after 3 days of coculturing.

### RNA isolation and RT-qPCR.

Total RNA from myofibroblasts was collected using the traditional TRIzol (Invitrogen, Thermo Fisher Scientific) method and quantified with NanoDrop (Thermo Fisher Scientific). cDNA was reverse transcribed from isolated RNA with PrimeScript RT Master Mix (Takara). RT-qPCR was performed with the ABI QuantStudio5 system. All RT-qPCR primer sequences are listed in Supplemental Table 17.

### Flow cytometric analysis.

The cells in each coculture system and single-cell suspension were harvested and divided into 2 separate tubes (Corning) for intracellular and intranuclear staining. For intracellular cytokine staining, cells were stimulated in vitro with cell stimulation cocktail (1:500, TNB-4975-UL100, Tonbo Biosciences) for 5 hours at 37°C with 5% CO_2_, followed by Ghost Dye Red 780 staining for 15 minutes and surface marker staining for 30 minutes in the dark. Next, cells were fixed and permeabilized with fixation and permeabilization buffer (eBioscience), after which they were stained with intracellular cytokine antibodies according to the manufacturer’s instructions. For intranuclear staining, the nuclear membranes were permeabilized with the Foxp3 Transcription Factor Staining Buffer Kit (TNB-0607-KIT, Tonbo Biosciences) prior to transcription factor staining. Flow cytometry was performed using an LSRFortessa instrument (BD Biosciences) and analyzed using FlowJo, version 10.

### Data and materials availability.

The raw data from the scRNA-Seq reported in this study have been deposited in the Genome Sequence Archive of the BIG Data Center at the Beijing Institution of Genomics, Chinese Academy of Sciences (accession no. HRA001006; https://bigd.big.ac.cn/gsa-human/browse/HRA001006). The codes to process and analyze data are publicly available in the GitHub repository (https://github.com/husimeng0717/scRNAseq-for-OSCC). Antibodies and reagents applied in this study are listed in Supplemental Table 18.

### Statistics.

For the survival analysis of the cohort in the TMAs (*n* = 142), a Kaplan-Meier curve for OS was generated, and a log-rank test was applied to assess differences between the TDO2^hi^ and TDO2^lo^ groups. Univariate Cox proportional models were first used to analyze associations between the clinical parameters and OS, among which the parameters with statistical significance were further included in a multivariate Cox regression analysis. The statistical methods and threshold for each analysis are described with the results or detailed in the figure legends or Methods. A 2-tailed Student’s *t* test was used to analyze differences in mean values between groups. All values are depicted as the mean ± SEM. A *P* value of less than 0.05 was considered significant. Multiple comparisons following 1-way ANOVA and Kruskal-Wallis test were performed for statistical analysis. Statistical analyses were performed using GraphPad Prism, version 9 (GraphPad Software).

### Study approval.

This study protocol for human tissue specimens was approved by the IRB of the Hospital of Stomatology, Sun Yat-sen University (Guangzhou, Guangdong, China) (approval no. KQEC-2020-09) and was conducted in agreement with Helsinki Declaration principles. Written informed consent was obtained from all study participants. All animal experiments were approved by and performed in accordance with the guidelines of the IRB and IACUC of Sun Yat-Sen University (approval no. 320727201100157128).

## Author contributions

ZW and FB conceived the study and directed and supervised the research. FB was responsible for bioinformatics analyses. ZW reviewed the histopathologic diagnoses. ZW, HL, and DW contributed to the enrollment of patients with OSCC or OLK and the collection of patient information. HL, WX, and DW performed tissue dissociation and single-cell suspension preparations and flow cytometric analysis. SH and HL helped with the design of the study and provided advice on the statistical analysis of scRNA-Seq data and scTCR-Seq data. SH, HL, XX, XMW, and W Dong contributed to data processing and figures generation. HL, WX, DW, ZS, YZ, W Dai, JG, SW, JF, and XYG conducted experiments and interpreted the data. All the authors contributed to manuscript preparation. SH wrote the original manuscript. ZW, FB, QC, and HL reviewed and edited the manuscript.

## Supplementary Material

Supplemental data

Supplemental tables 1-18

Supplemental video 1

Supplemental video 2

## Figures and Tables

**Table 1:**
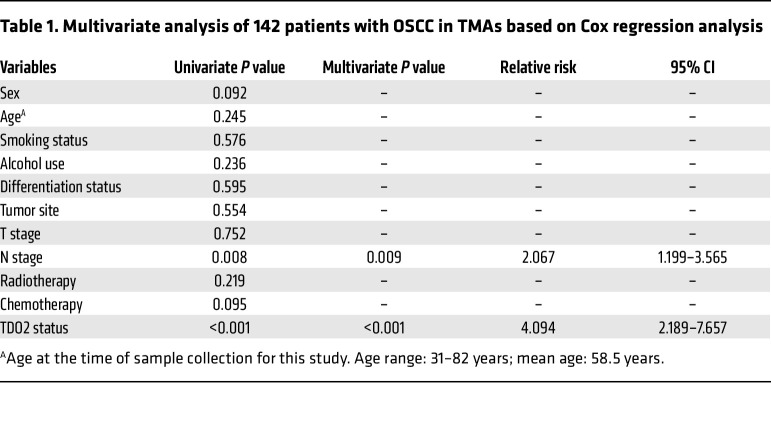
Multivariate analysis of 142 patients with OSCC in TMAs based on Cox regression analysis

## Version 1 08/16/2022

In-Press Preview

## Version 2 10/03/2022

Electronic publication

## References

[1] Sung H (2021). Global cancer statistics 2020: GLOBOCAN estimates of incidence and mortality worldwide for 36 cancers in 185 countries. CA Cancer J Clin.

[2] Chi AC (2015). Oral cavity and oropharyngeal squamous cell carcinoma--an update. CA Cancer J Clin.

[3] Yardimci G (2014). Precancerous lesions of oral mucosa. World J Clin Cases.

[4] Evren I (2020). Annual malignant transformation rate of oral leukoplakia remains consistent: a long-term follow-up study. Oral Oncol.

[5] Ribas A, Wolchok JD (2018). Cancer immunotherapy using checkpoint blockade. Science.

[6] Schoenfeld JD (2020). Neoadjuvant nivolumab or nivolumab plus ipilimumab in untreated oral cavity squamous cell carcinoma: a phase 2 open-label randomized clinical trial. JAMA Oncol.

[7] Ferris RL (2016). Nivolumab for recurrent squamous-cell carcinoma of the head and neck. N Engl J Med.

[8] Burtness B (2019). Pembrolizumab alone or with chemotherapy versus cetuximab with chemotherapy for recurrent or metastatic squamous cell carcinoma of the head and neck (KEYNOTE-048): a randomised, open-label, phase 3 study. Lancet.

[9] Segal NH (2019). Safety and efficacy of durvalumab in patients with head and neck squamous cell carcinoma: results from a phase I/II expansion cohort. Eur J Cancer.

[10] Harper J, Sainson RC (2014). Regulation of the anti-tumour immune response by cancer-associated fibroblasts. Semin Cancer Biol.

[11] Wang J (2016). The role of cancer-associated fibroblasts in esophageal cancer. J Transl Med.

[12] Garcia-Honduvilla N (2017). High sensitivity of human adipose stem cells to differentiate into myofibroblasts in the presence of C. aspersa egg extract. Stem Cells Int.

[13] Chen X, Song E (2019). Turning foes to friends: targeting cancer-associated fibroblasts. Nat Rev Drug Discov.

[14] Li X (2019). CXCL12/CXCR4 pathway orchestrates CSC-like properties by CAF recruited tumor associated macrophage in OSCC. Exp Cell Res.

[15] Li J (2020). Small extracellular vesicle-bound vascular endothelial growth factor secreted by carcinoma-associated fibroblasts promotes angiogenesis in a bevacizumab-resistant manner. Cancer Lett.

[16] Jiang H (2020). Development of resistance to FAK inhibition in pancreatic cancer is linked to stromal depletion. Gut.

[17] Dominguez CX (2020). Single-cell RNA sequencing reveals stromal evolution into LRRC15^+^ myofibroblasts as a determinant of patient response to cancer immunotherapy. Cancer Discov.

[18] Costa A (2018). Fibroblast heterogeneity and immunosuppressive environment in human breast cancer. Cancer Cell.

[19] Zilionis R (2019). Single-cell transcriptomics of human and mouse lung cancers reveals conserved myeloid populations across individuals and species. Immunity.

[20] Zhang L (2018). Lineage tracking reveals dynamic relationships of T cells in colorectal cancer. Nature.

[21] Puram SV (2017). Single-cell transcriptomic analysis of primary and metastatic tumor ecosystems in head and neck cancer. Cell.

[22] Cillo AR (2020). Immune landscape of viral- and carcinogen-driven head and neck cancer. Immunity.

[23] Pabbisetty SK (2014). KLF2 is a rate-limiting transcription factor that can be targeted to enhance regulatory T-cell production. Proc Natl Acad Sci U S A.

[24] Luo CT (2016). Graded Foxo1 activity in Treg cells differentiates tumour immunity from spontaneous autoimmunity. Nature.

[25] Miller BC (2019). Subsets of exhausted CD8 ^+^ T cells differentially mediate tumor control and respond to checkpoint blockade. Nat Immunol.

[26] Hudson WH (2019). Proliferating transitory T cells with an effector-like transcriptional signature emerge from PD-1 ^+^ stem-like CD8 ^+^ T cells during chronic infection. Immunity.

[27] Cheng S (2021). A pan-cancer single-cell transcriptional atlas of tumor infiltrating myeloid cells. Cell.

[28] Zhang Q (2019). Landscape and dynamics of single immune cells in hepatocellular carcinoma. Cell.

[29] Kuppe C (2021). Decoding myofibroblast origins in human kidney fibrosis. Nature.

[30] Buechler MB (2021). Cross-tissue organization of the fibroblast lineage. Nature.

[31] Si Z (2019). Adipose-derived stem cells: sources, potency, and implications for regenerative therapies. Biomed Pharmacother.

[32] Zavala G (2019). Differentiation of adipose-derived stem cells to functional CD105 ^neg^ CD73 ^low^ melanocyte precursors guided by defined culture condition. Stem Cell Res Ther.

[33] Mildmay-White A, Khan W (2017). Cell surface markers on adipose-derived stem cells: a systematic review. Curr Stem Cell Res Ther.

[34] Platten M (2014). Cancer immunotherapy by targeting IDO1/TDO and their downstream effectors. Front Immunol.

[35] Ohlund D (2017). Distinct populations of inflammatory fibroblasts and myofibroblasts in pancreatic cancer. J Exp Med.

[36] Chen X (2021). CD8^+^ T effector and immune checkpoint signatures predict prognosis and responsiveness to immunotherapy in bladder cancer. Oncogene.

[37] Raines EW (1989). Interleukin-1 mitogenic activity for fibroblasts and smooth muscle cells is due to PDGF-AA. Science.

[38] Opitz CA (2011). An endogenous tumour-promoting ligand of the human aryl hydrocarbon receptor. Nature.

[39] Pilotte L (2012). Reversal of tumoral immune resistance by inhibition of tryptophan 2,3-dioxygenase. Proc Natl Acad Sci U S A.

[40] Thorsson V (2018). The immune landscape of cancer. Immunity.

[41] Mandal R (2016). The head and neck cancer immune landscape and its immunotherapeutic implications. JCI Insight.

[42] Ohman J (2015). Presence of CD3-positive T-cells in oral premalignant leukoplakia indicates prevention of cancer transformation. Anticancer Res.

[43] Dong Y (2021). PD-1 blockade prevents the progression of oral carcinogenesis. Carcinogenesis.

[44] Schietinger A (2016). Tumor-specific T cell dysfunction is a dynamic antigen-driven differentiation program initiated early during tumorigenesis. Immunity.

[45] Molgora M (2020). TREM2 modulation remodels the tumor myeloid landscape enhancing anti-PD-1 immunotherapy. Cell.

[46] Katzenelenbogen Y (2020). Coupled scRNA-seq and intracellular protein activity reveal an immunosuppressive role of TREM2 in cancer. Cell.

[47] Salmon H (2012). Matrix architecture defines the preferential localization and migration of T cells into the stroma of human lung tumors. J Clin Invest.

[48] Mariathasan S (2018). TGFβ attenuates tumour response to PD-L1 blockade by contributing to exclusion of T cells. Nature.

[49] Gorchs L (2019). Human pancreatic carcinoma-associated fibroblasts promote expression of co-inhibitory markers on CD4^+^ and CD8^+^ T-cells. Front Immunol.

[50] Ene-Obong A (2013). Activated pancreatic stellate cells sequester CD8^+^ T cells to reduce their infiltration of the juxtatumoral compartment of pancreatic ductal adenocarcinoma. Gastroenterology.

[51] Elyada E (2019). Cross-species single-cell analysis of pancreatic ductal adenocarcinoma reveals antigen-presenting cancer-associated fibroblasts. Cancer Discov.

[52] Yang X (2016). FAP promotes immunosuppression by cancer-associated fibroblasts in the tumor microenvironment via STAT3-CCL2 signaling. Cancer Res.

[53] Kraman M (2010). Suppression of antitumor immunity by stromal cells expressing fibroblast activation protein-alpha. Science.

[54] Feig C (2013). Targeting CXCL12 from FAP-expressing carcinoma-associated fibroblasts synergizes with anti-PD-L1 immunotherapy in pancreatic cancer. Proc Natl Acad Sci U S A.

[55] Lieu EL (2020). Amino acids in cancer. Exp Mol Med.

[56] Cheong JE, Sun L (2018). Targeting the IDO1/TDO2-KYN-AhR pathway for cancer immunotherapy — challenges and opportunities. Trends Pharmacol Sci.

[57] Wang Z (2019). Syngeneic animal models of tobacco-associated oral cancer reveal the activity of in situ anti-CTLA-4. Nat Commun.

[58] Wheeler SE (2014). Enhancement of head and neck squamous cell carcinoma proliferation, invasion, and metastasis by tumor-associated fibroblasts in preclinical models. Head Neck.

