# Supplementary material for: TDO2+ myofibroblasts mediate immune suppression in malignant transformation of squamous cell carcinoma
Source: J Clin Invest. 2022 Oct 3;132(19):e157649. doi: 10.1172/JCI157649 (PMC9525123; doi:10.1172/JCI157649)
Supplement: Supplemental data [file jci-132-157649-s089.pdf]

## Supplementary figures and figure legends

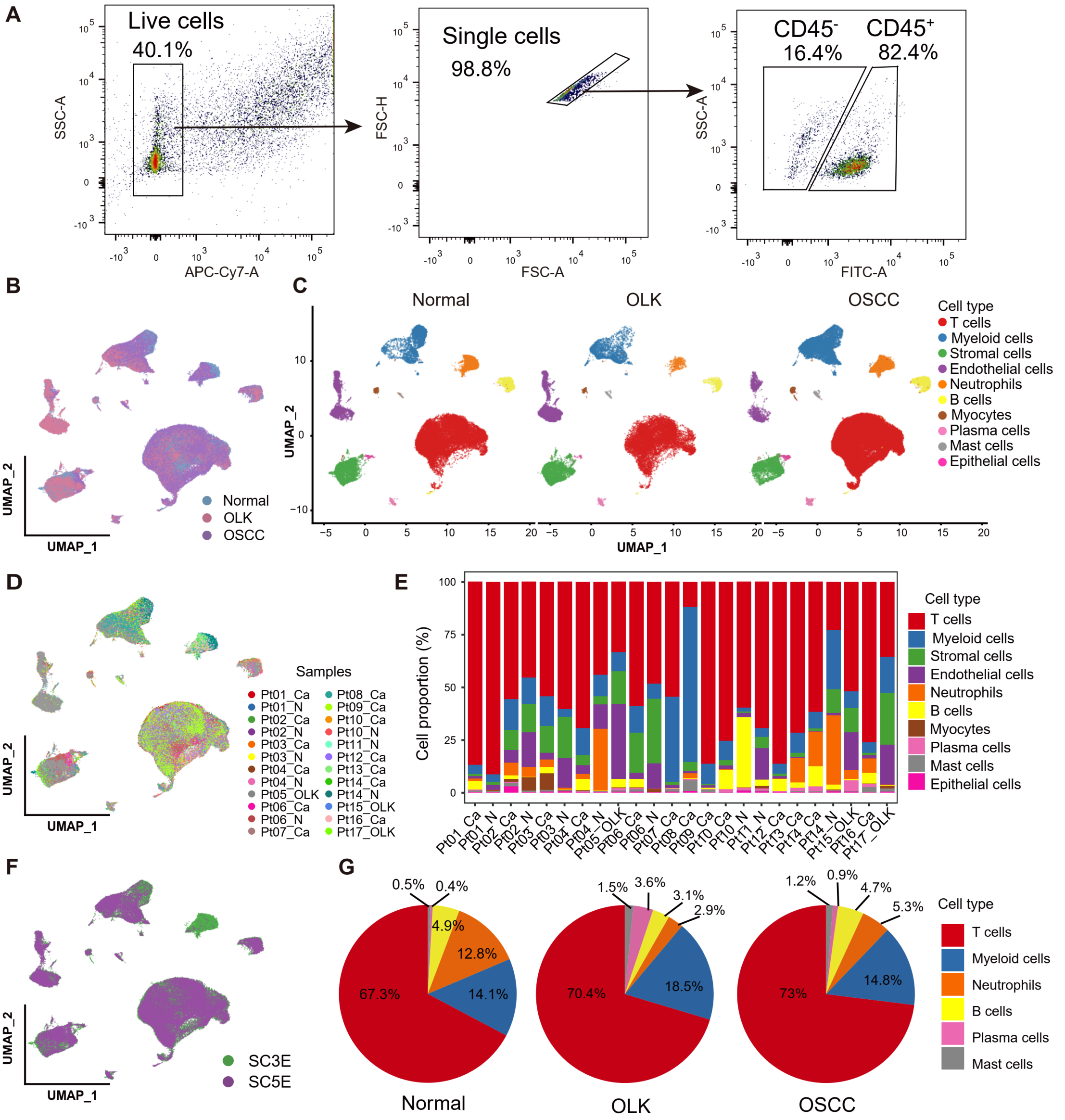


**Supplementary Figure 1. scRNA-seq analysis of 13 OSCC, 3 OLK and 8 adjacent normal samples from 17 patients.**

(**A**) The proportions of CD45$^{+}$ (immune) and CD45$^{-}$ (non-immune) cells among live cells based on FACS of the tumor tissue cell suspension after dissociation with a tumor dissociation kit. (**B**) UMAP colored to indicate normal, OLK and OSCC tissues. **(C)** UMAP plots showing the cellular compositions of normal, OLK and OSCC tissues. **(D)** UMAP plot showing the distribution of all cells in all 24 samples. Pt, patients; Ca, OSCC tissue; OLK, leukoplakia; N, adjacent normal tissue. **(E)** Bar plot showing the distributions of major cell types among 24 samples. **(F)** Cells were colored according to the single-cell reagent kit. SC3E indicates the single-cell 3′ reagent kit, and SC5E indicates the single-cell 5′ reagent kit. **(G)** Pie charts showing the percentages of each major immune cell type among the total immune cells in normal, OLK and OSCC tissues.

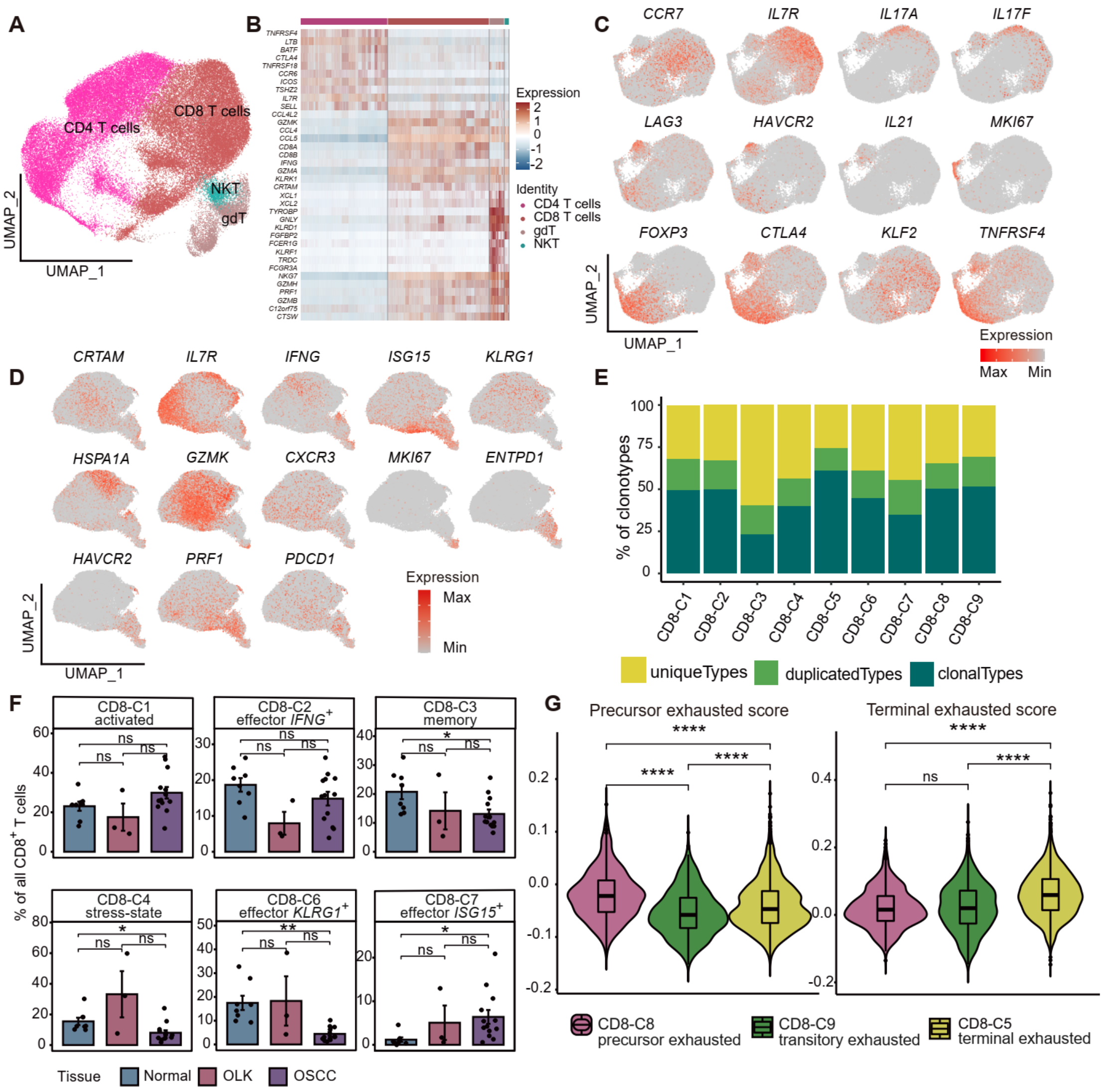


**Supplementary Figure 2. Cellular and functional characterization of $CD4^+$ and $CD8^+$ T cells.** (**A**) UMAP plot showing the distribution of the main lineages in T cells. The color represents the T cell lineage. (**B**) Heatmap showing the top 10 upregulated genes in each subset of T cells. Rows represent genes and columns represent cells. In the heatmap, red indicates high expression, while blue indicates low expression. Each color in the bar above the heatmap represents a T cell subset. **(C)** UMAP plot showing the expression levels of specifically expressed genes in $CD4^+$ T cell subsets. The red color indicates the higher expression level. Min indicates the minimum expression level, and Max indicates the maximum expression level. **(D)** UMAP plot showing the expression levels of specifically expressed genes in $CD8^+$ T cell subsets. **(E)** Bar plots showing the percentages of TCR expanded clonotypes in the $CD8^+$ T cell subsets. **(F)** Bar plots showing the percentages of 6 $CD8^+$ T cell subsets among the total $CD8^+$ T cells in adjacent normal, OLK and OSCC tissues. **(G)** Violin plot showing the scores of the precursor and terminal exhaustion modules in terminal exhausted $CD8^+$ T cells (CD8-C5), precursor exhausted $CD8^+$ T cells (CD8-C8) and transitory exhausted $CD8^+$ T cells (CD8-C9). Each color represents a cell type. The center line of the box represents the median value, the upper and lower limits of the box represent the 25th and 75th percentile points. (**F** and **G**) Kruskal-Wallis test followed by Bonferroni's multiple-comparison test; *, $P < 0.05$; **, $P < 0.01$; ****, $P < 0.0001$; ns, no significant difference.

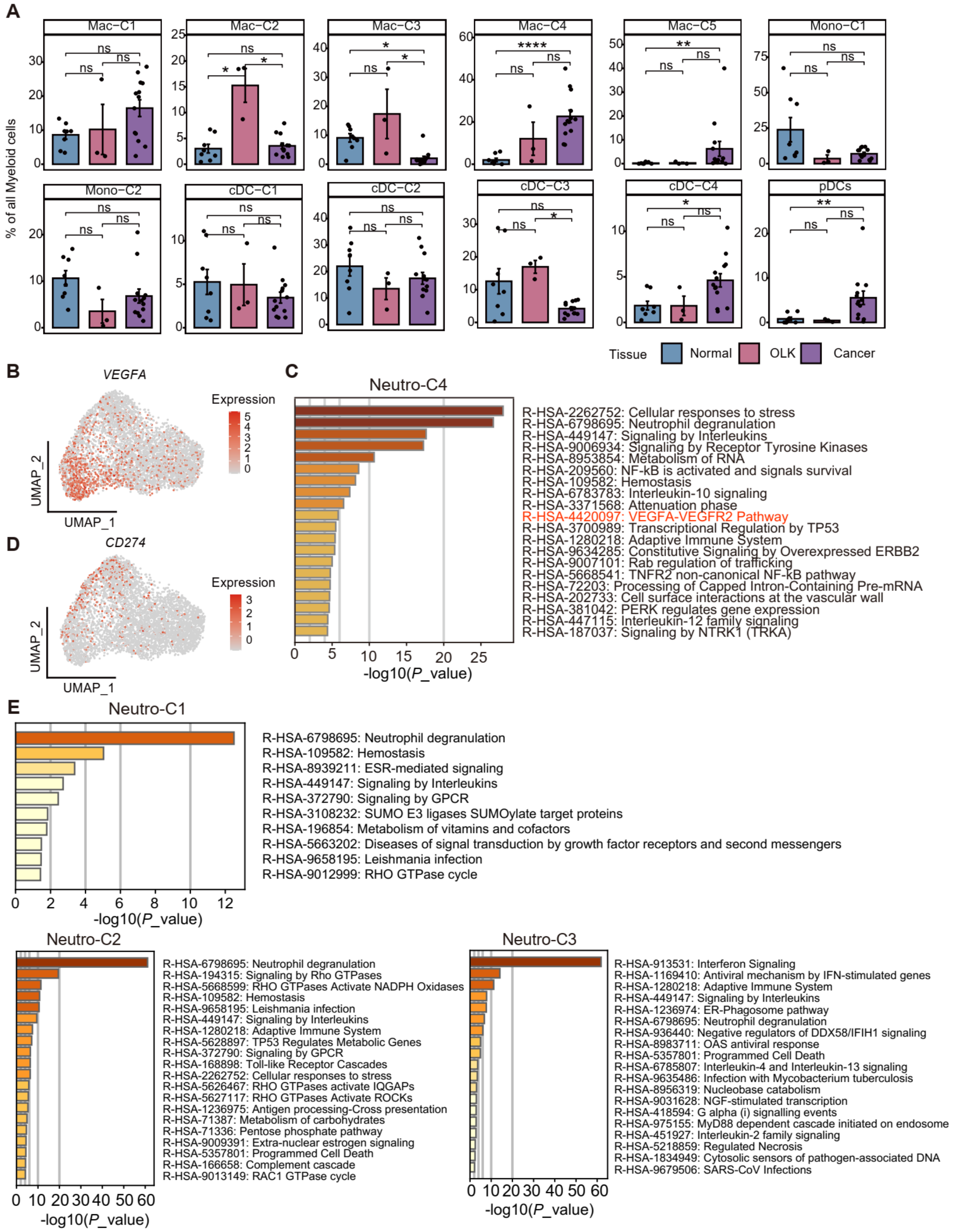


**Supplementary Figure 3. Percentages of myeloid cell subsets and characterization of neutrophil subsets.**

(**A**) Bar plots showing the percentage of myeloid cell subsets among the total myeloid cells in adjacent normal, OLK and OSCC tissues. Kruskal-Wallis test followed by Bonferroni's multiple-comparison test; *, $P < 0.05$; **, $P < 0.01$; ****, $P < 0.0001$; ns, no significant difference. (**B**) UMAP plot showing the expression levels of *VEGFA* in neutrophil subsets. **(C)** Bar plot showing the results of the enrichment analysis of the set of genes highly expressed in Neutro-C4 in the Reactome database, with the horizontal coordinate representing -log10 (*P*-value). **(D)** UMAP plot showing the expression levels of *CD274* in neutrophil subsets. **(E)** Bar plot showing the results of the enrichment analysis of the set of genes highly

expressed in Neutro-C1-C3 in the Reactome database, with the horizontal coordinate representing -log10 (*P*-value). (**C** and **E**) Hypergeometric distribution; $P < 0.01$.

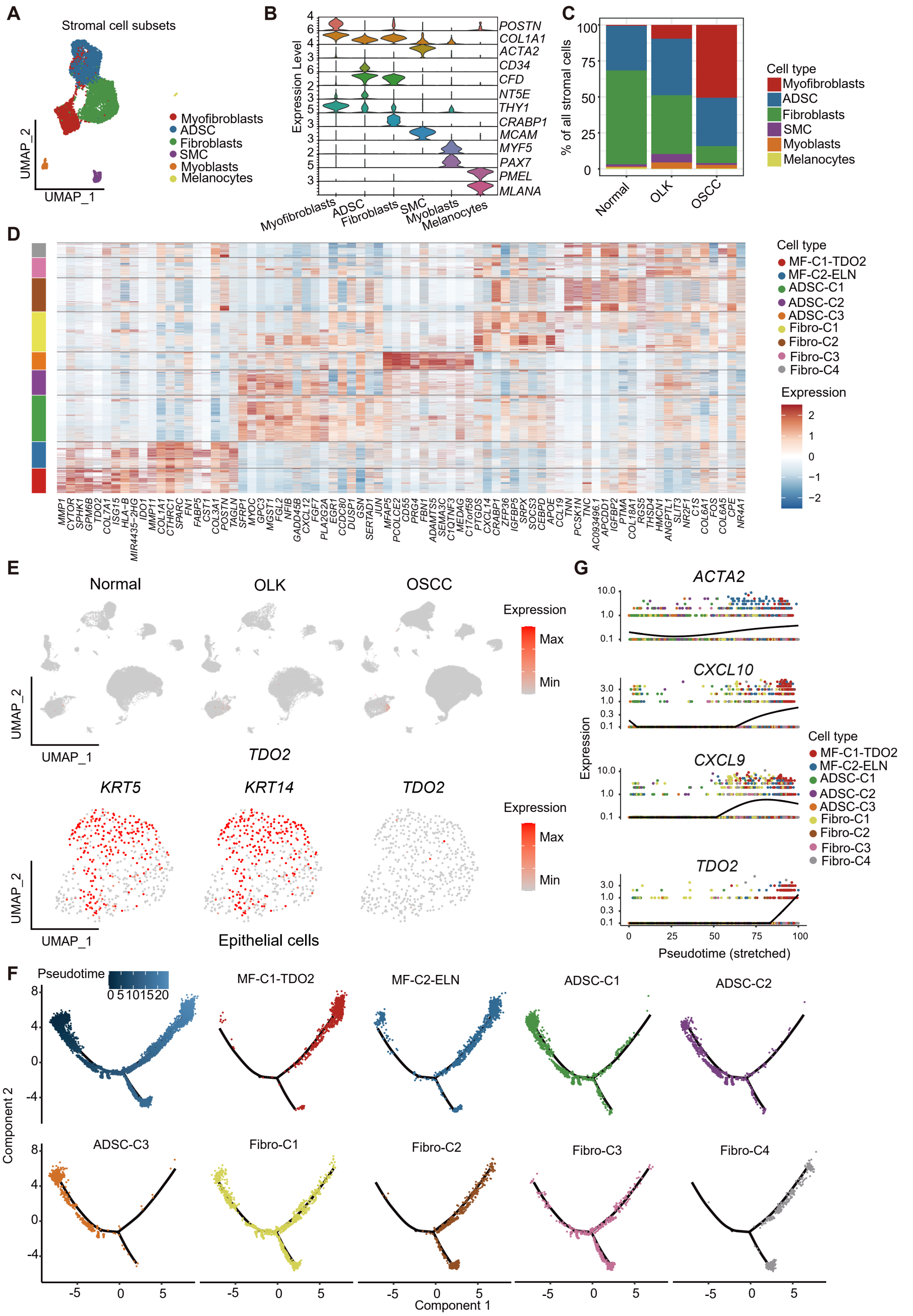


**Supplementary Figure 4. Characterization and pseudotime trajectory of stromal cell subsets.** (**A**) UMAP plot showing the distribution of major cell types among all stromal cells. Each color represents a major cell type. (**B**) Violin plot showing the expression levels of cell type markers in each major stromal cell subset. Each color represents a gene. **(C)** Stacked histogram showing the percentages of stromal cell subsets among total stromal cells of adjacent normal, OLK and OSCC tissues. **(D)** Heatmap showing the expression levels of the top 10 highly expressed marker genes in each subset of

ADSC-Fibro-MF cells. Rows represent genes and columns represent cells; each color of the bar above the heatmap represents a cell subset. Red indicates high expression, and blue indicates low expression. **(E)** UMAP plot showing the expression levels of *TDO2* in all cells of adjacent normal, OLK and OSCC tissues (upper) and the expression levels of *KRT5, KRT14* and *TDO2* in epithelial cells of all tissues (lower). **(F)** The putative differentiation directions inferred from the pseudotime analysis among ADSC-Fibro-MF cells (upper left). Darker shading indicates a lower pseudotime value. The branch distribution of each subset is shown. **(G)** The expression levels of *ACTA2*, *CXCL10*, *CXCL9* and *TDO2* from the results of the pseudotime analysis of ADSC-Fibro-MF cells. Each color represents a cell subset.

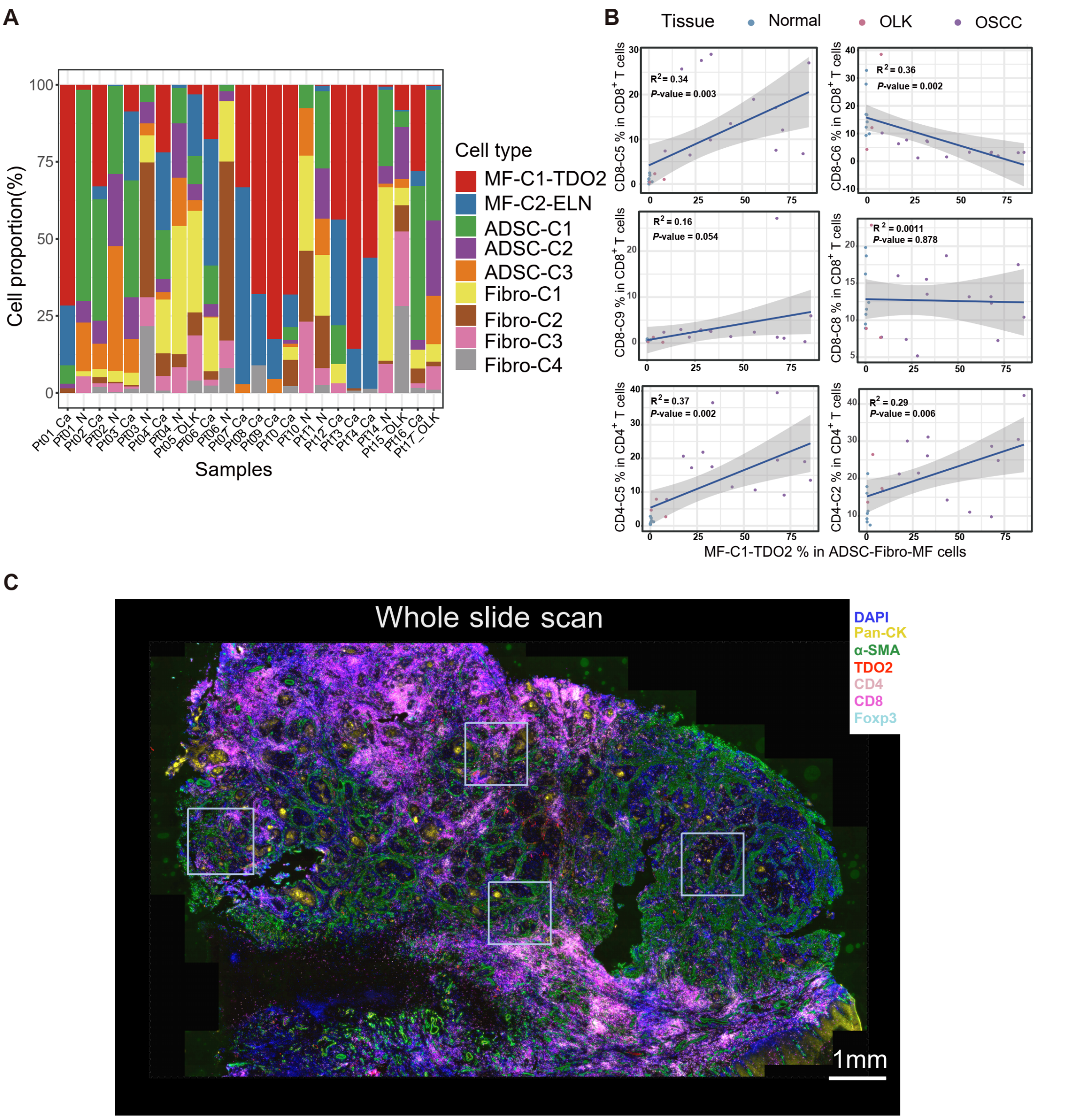


**Supplementary Figure 5. Relative proportions of ADSC-Fibro-MF cells and whole-side scan image of a mIHC slide.**

(**A**) Bar plot showing the distribution of ADSC-Fibro-MF cell subsets among 24 samples. Pt, patients; Ca, OSCC tissue; OLK, leukoplakia; N, adjacent normal tissue. (**B**) Scatter plot showing the correlation analysis between the relative abundance of MF-C1-TDO2 myofibroblasts and some T cell subsets. Each point color represents a tissue type. **(C)** A whole-side scan image of a multiplex immunohistochemical staining (mIHC) slide of Pt10_Ca on the Vectra platform. We captured 4 fields per slide, resulting in a total of 40 fields from 10 whole-side scan images for further quantitative analysis. A white box represents a 10× high-powered field. Scale bar: 1 mm.

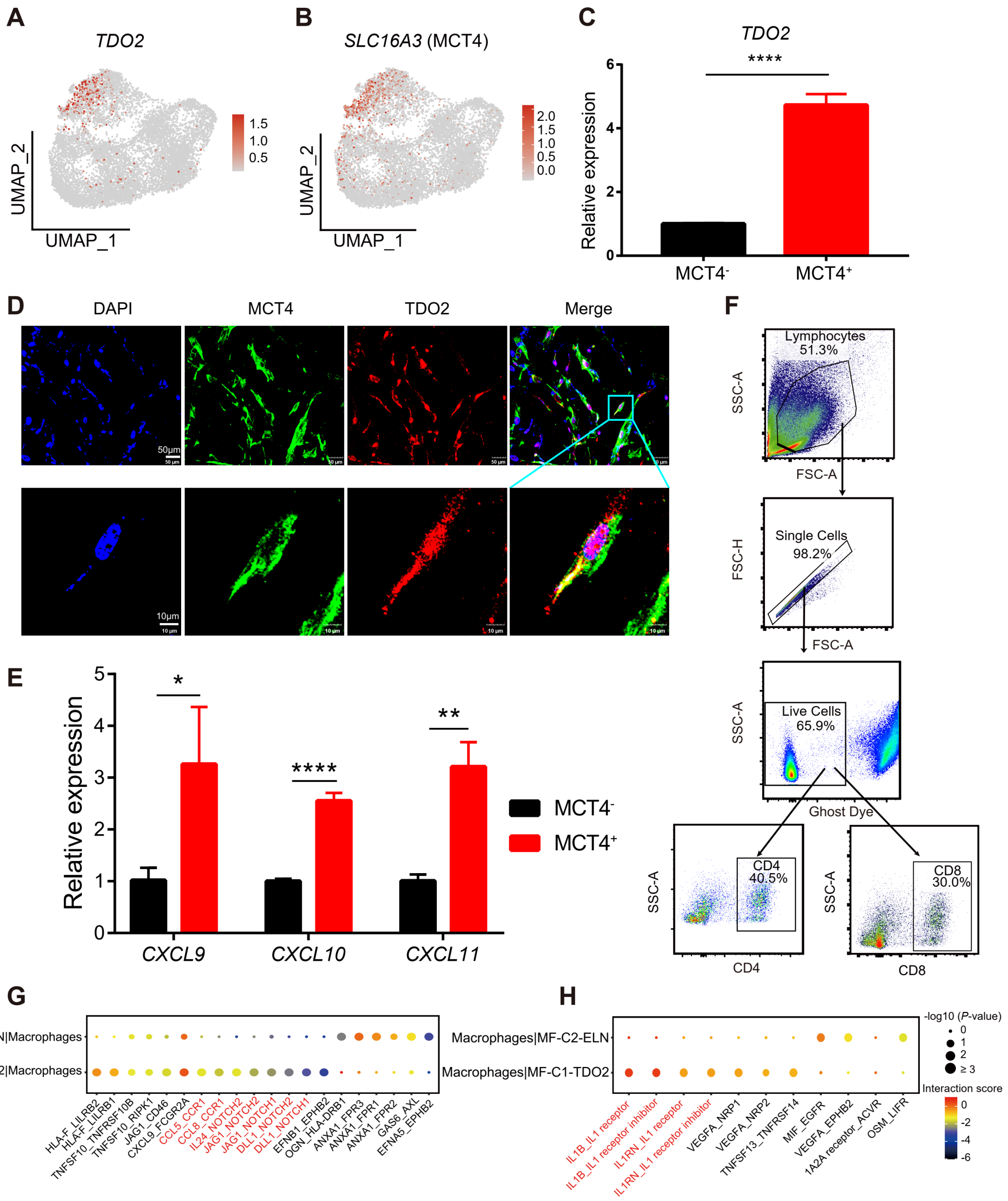


**Supplementary Figure 6. MCT4 is specifically expressed on TDO2⁺ myofibroblasts.**

(**A** and **B**) UMAP plot showing the expression levels of (A) *TDO2* and (B) *SLC16A3* (encoding MCT4) in ADSC-Fibro-MF cells. (**C**) RT-qPCR results showing the relative expression level of *TDO2* in MCT4$^{+}$ myofibroblasts compared to the MCT4$^{-}$ myofibroblasts. (**D**) Immunofluorescence imaging results showing the spatial localization of TDO2 (red) and MCT4 (green) in myofibroblasts isolated from OSCC; scale bar (upper): 50 μm; scale bar (lower): 10 μm. (**E**) RT-qPCR results showing the relative expression level of *CXCL9/10/11* in MCT4$^{+}$ myofibroblasts compared to the MCT4$^{-}$ myofibroblasts. **(F)** The gating strategy for flow cytometry of CD4$^{+}$ and CD8$^{+}$ T cells. (**G** and **H**) Dot plot showing the interaction intensity between myofibroblasts (MF-C2-ELN and MF-C1-TDO2) and macrophages according to CellPhoneDB analysis. The dot color represents the interaction score and the dot size represents the -log (P-value). (G) The interaction of ligand of myofibroblasts with receptor of macrophages. (H) The interaction of ligand of macrophages with receptor of myofibroblasts.

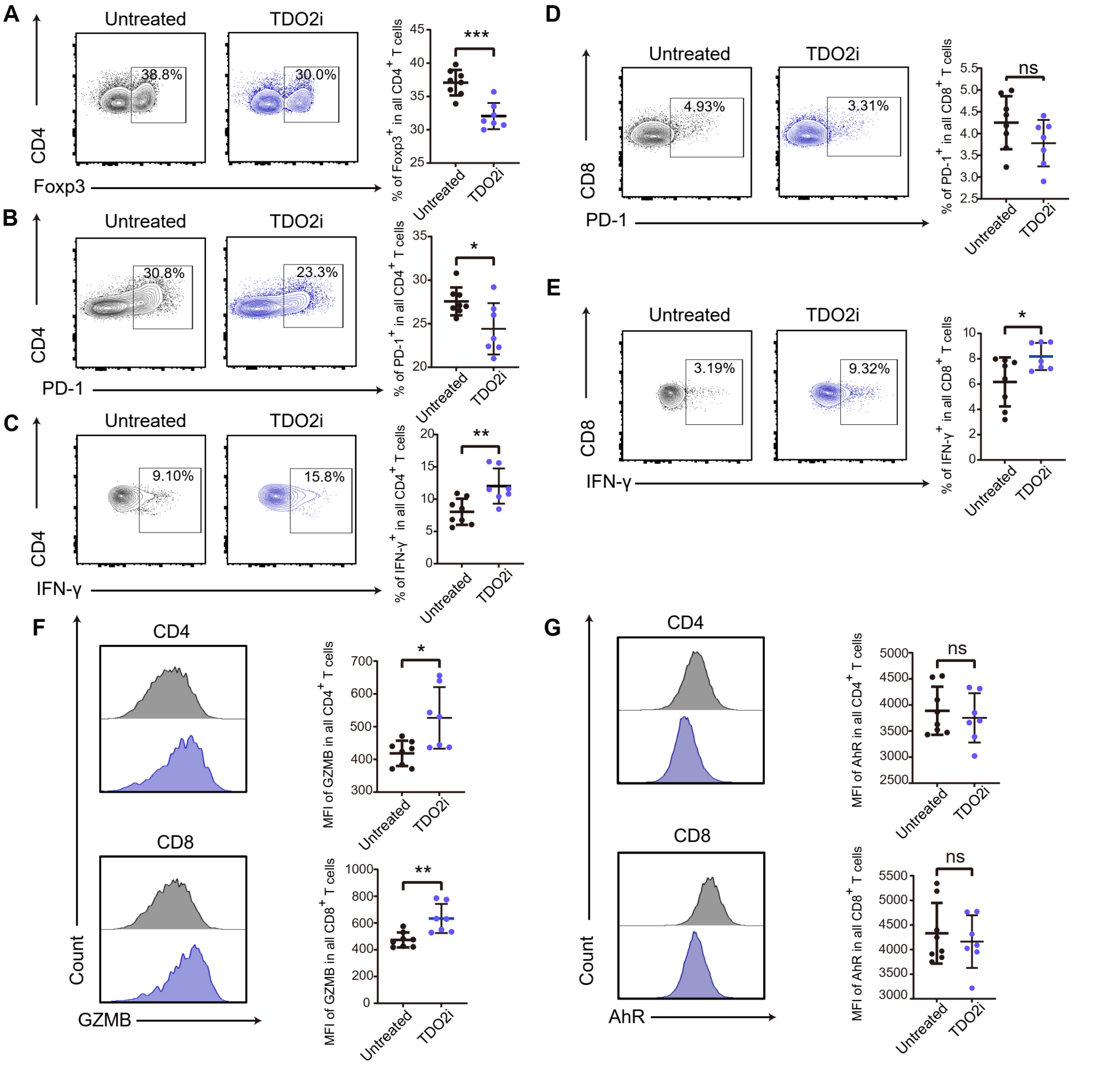


**Supplementary Figure 7. Inhibition of TDO2 attenuated the inhibitory states of T cells in draining lymph nodes (dLN) in the 4NQO-induced carcinogenic murine mode.**

**(A-C)** Representative flow cytometry images (left) and statistical results (right) showing the proportions of (A) Foxp3$^+$, (B) PD-1$^+$ and (C) IFN-γ$^+$ CD4$^+$ T cells from dLN samples from the TDO2i and untreated groups. **(D** and **E)** Representative flow cytometry images (left) and statistical results (right) showing the proportions of (D) PD-1$^+$ and (E) IFN-γ$^+$ CD8$^+$ T cells from dLN samples from the TDO2i and untreated groups. **(F)** Representative flow cytometry images (left) and statistical results (right) showing the median fluorescence intensity (MFI) of GZMB in CD4$^+$ (upper) and CD8$^+$ (lower) T cells from dLN samples from the TDO2i and untreated groups. **(G)** Representative flow cytometry images (left) and statistical results (right) showing the MFI of AhR between CD4$^+$ (upper) and CD8$^+$ (lower) T cells from the TDO2i and untreated groups. (**A-G**) *, $P < 0.05$; **, $P < 0.01$; ***, $P < 0.001$; ns, no significant difference; 2-tailed Student's $t$ test.

## Supplemental Methods

**Tissue dissociation and single-cell suspensions**

Fresh samples were trimmed, washed with Dulbecco's phosphate-buffered saline (D-PBS; ThermoFisher Scientific, Waltham, MA), minced, and dissociated using a Human Tumor Dissociation Kit (Miltenyi Biotec, Bergisch Gladbach, Germany) according to the manufacturer guidelines. Cell suspensions were filtered by a 70 mm nylon mesh filter (ThermoFisher Scientific), and dissociated cells were pelleted and lysed with BD Pharm Lyse (BD Biosciences, Franklin Lakes, NJ). Samples were then stained with Zombie Fixable Viability Dye (Biolegend, San Diego, CA) at a 1:100 dilution for 15 minutes at room temperature (RT), and washed with PBS with 2% fetal bovine serum (FBS; ThermoFisher Scientific). Cells were then stained for sorting by incubation with FITC-conjugated mouse anti-human CD45 (clone: HI30; Biolegend) at a 1:100 dilution for 30 minutes at 4 °C, spun down at $500 \times g$ for 5 minutes, washed with cold PBS, and re-suspended with PBS for single-cell sorting. Live cells were sorted from 100 μm flow cytometry nozzle by BD FACS AriaFusion (BD Biosciences) and the proportions of $CD45^+$ cells were recorded. Single cells were collected in pure FBS. Then all the cells were processed in less than one hour after sorting. Sorted cells were washed and re-suspended in cold PBS (containing 0.04% BSA) at $7–12 \times 10^5$ cells/mL before loading into a Chromium Single Cell Controller.

**Preparation of scRNA-seq libraries and sequencing**

Single cell transcriptome sequencing was performed using the droplet-based 10X Genomics platform. Briefly, the single cell suspension was added to each channel in a Chromium Single Cell Controller, and cells were captured using Gel Bead Kit V3 or

V2 reagents based on microfluidic technology. Gel beads in emulsion (GEM) were encapsulated in oil droplets, with each GEM containing a cell, a unique cell barcode, a unique molecular identifier (UMI) and a reverse transcription reaction mixture (RT-qPCR). The captured cells were lysed in GEM. The RNA released from the cells was processed by reverse transcription in a single GEM with the addition of a barcode and UMI, with each cell possessing a unique barcode and each gene in the cell possessing a unique UMI. Subsequent reverse transcription was performed at 53 °C for 45 min, followed by 5 min at 85 °C, and then the temperature was maintained at 4 °C. The resulting cDNA was amplified and then assessed for quality using an Agilent 4200 according to the manufacturers instructions. For 14 samples, single cell transcriptome libraries were constructed using Chromium Single Cell 3′ Library kits. For 10 samples, single cell transcriptome libraries were constructed using 5′ Library kits, and TCR-enriched libraries were generated with aliquots from each of the aforementioned cDNAs using the Chromium Single Cell V(D)J Enrichment kit. The libraries were sequenced using the Illumina NovaSeq 6000 sequencing platform.

**Raw data processing and quality control of scRNA-seq data**

Raw sequencing data from the 10x Genomics platform were converted to fastq format using ‘CellRanger mkfastq’ (v.4.0.0). Next, scRNA-seq reads were aligned to the GRCh38 reference genome using ‘CellRanger count’ (v4.0.0).

To analyze the results from the above pipeline using ‘CellRanger’, we used the Seurat package (v.3.2.2) in R (v.3.6.3) to visualize the scRNA-seq data (1). Our initial dataset contained 153,035 cells. Stringent data quality control was conducted during

the downstream analysis. Only genes detected in at least 0.1% of cells were retained. We filtered the cells with the following parameters to exclude outliers: maximum percentage mito=20%, maximum number of UMIs=60,000, minimum number of nGene=300, and maximum number of nGene=7,500. Then, double cell scoring was performed using the R package scDblFinder (v.1.4.0) to remove cells that were considered to be double cells in each sample (2).

After discarding poor-quality cells, a total of 131,702 cells were retained for downstream analysis. To normalize the library size effect in each cell, we scaled UMI counts using scale.factor=10,000. Following log-transformation of the data, other factors, including "percent.mt" and "nCount_RNA", were corrected for variation regression using the ScaleData function. To eliminate batch effects, the top 3,000 variable genes were extracted for run fastMNN based on the mutual nearest neighbors (MNN) method that was included in Seurat (3). We performed PCA using variably expressed genes under the "mnn" assay mode. The top 30 PCs were used for subsequent clustering and uniform manifold approximation and projection (UMAP) visualization. Forty initial clusters were identified with the FindClusters function using shared nearest neighbor modularity optimization with the clustering resolution set to 1.5.

**Cell type annotation**

We first searched for the top differential markers for each identified cluster/sub-cluster using the FindAllMarkers function. The test method used for FindAllMarkers was the Wilcoxon rank sum test. For each cell type, we used multiple cell-type-

specific/enriched marker genes that were previously described in the literature to determine cellular identity. These include, but were not limited to, *CD3E*, *CD3D* and *CD3G* for T cells (4); *LYZ*, *CD14* and *C1QB* for myeloid cells (5); *DCN*, *COL1A1* and *COL3A1* for stromal cells (6); *TM4SF1*, *PECAM1* and *VWF* for endothelial cells (7); *CXCL8*, *G0S2* and *CSF3R* for neutrophils (8); *MS4A1*, *CD79A* and *CD79B* for B cells (9); *ACTA1*, *MYL1* and *MYH2* for myocytes (10); *MZB1*, *DERL3* and *IGKC* for plasma cells (11); *TPSB2*, *TPSAB1* and *CPA3* for mast cells (12); and *KRT14*, *KRT5* and *KRT17* for epithelial cells (10). Cells with expression of double-lineage genes, such as *LYZ*$^+$*DCN*$^+$ cells and *LYZ*$^+$*VWF*$^+$ cells, were excluded to eliminate potential doublet capture bias. We then arranged all of the identified cell types into 10 major cell sets based on their expression profiles, lineages, and functions.

**Subclustering of T cells, myeloid cells, neutrophils and stromal cells**

For major cell types (T cells, myeloid cells, neutrophils and stromal cells), cells were extracted from the integrated dataset first. Next, we performed PCA using the variably expressed genes for each of the major cell type objects under the “mnn” assay mode. The top 30 PCs were used for subsequent clustering and UMAP visualization. The FindClusters function of the R package Seurat was utilized with suitable resolution to identify sub-clusters within major cell types. For T cells, we removed the low quality clusters again, as their majority of cells having greater than 15% mitochondrial RNA, under 1,000 detected transcripts, or under 400 unique genes.

**Scored cell state signature**

Precursor exhausted and terminal exhausted modules of CD8$^+$ T cells were scored using

the AddModuleScore function of the R package Seurat. The precursor exhausted and terminal exhausted signatures were derived from previous studies (13). The precursor exhausted signature consisted of the genes *COLQ*, *OAF*, *F2RL1*, *GZMM*, *AQP3*, *GALNT14*, *SLC2A6*, *FAM81A*, *SAMD3*, *P2RX7*, *SH3BP5*, *TBC1D4*, *SSPO*, *IL18*, *LRIG1*, *TESPA1*, *SH2B3*, *FAM160A1*, *S1PR5*, *KLF3*, *CD83*, *XCL1*, *CXXC5*, *TNFRSF13B*, *ST8SIA1*, *SELL*, *DHRS3*, *DTX1*, *CD40LG*, *KCNMB1*, *WNT10A*, *SOSTDC1*, *SYNPO*, *TREML2*, *LIF*, *S1PR1*, *TNFSF8*, *TNFSF14*, *ART3*, *MAPK11*, *HECTD2*, *TNFRSF25*, *CD22*, *SLAMF6*, *ID3*, *DAPL1*, *CXCR5*, *AFF3*, *TCF7*, and *CCR6*. The terminal exhausted signature consisted of the genes *DSC2*, *RASD2*, *LTF*, *CCR1*, *HTRA3*, *LGI2*, *MGAT3*, *GLIS1*, *FCRL6*, *HAVCR2*, *CD244*, *RASSF6*, *GZMB*, *FILIP1*, *CDKN2A*, *ADAM8*, *CDH17*, *FCER1G*, *EPDR1*, *CHL1*, *IL1R2*, *CCL3*, *SPP1*, *ACOXL*, *ENTPD1*, *NEB*, *LY6G5B*, *UPP1*, *AOAH*, *MREG*, *P2RY14*, *ADORA3*, *EPAS1*, *PLXND1*, *CDKN1A*, *NPNT*, *FGL2*, *ASB2*, *PPP1R3B*, *IL10*, *GPR35*, *ADRB1*, *LAT2*, *RASL12*, *SLC13A3*, *SLC16A10*, *PRF1*, *MYO10*, *CD14*, and *CDKN2B*. For the ADSC-Fibro-MF subsets, we scored AhR activation module, which consisted of the genes *IDO1*, *IDO2*, *TDO2*, *IL4I1*, *KYNU*, and *AHR* (14, 15). The module scores were calculated using the default parameters. The R package ggplot2 (v3.3.2) was used to visualize the results.

**Inferring the differentiation trajectories of $CD4^+$ and $CD8^+$ T cells using scVelo**

To infer the differentiation trajectories of $CD4^+$ and $CD8^+$ T cells, we used scVelo (v0.2.2) to analyze the RNA velocity in individual cells (16). scVelo performs calculations of transcriptional dynamics based on the ratio of “unspliced” pre-mRNA and “spliced” mRNA of each gene in each cell to obtain a gene expression change rate

(17). Application of this method allows researchers to estimate in which direction the gene expression profile of a given cell might switch, inferring possible developmental relationships between different cell types in a tissue sample.

Briefly, we used the Python module 'velocyto run10x' (v0.17.17) to analyze the BAM files (the output files from 'CellRanger count') to obtain loom files, and the loom files of all samples were merged by the Python module loompy (v3.0.6). Next, we integrated Seurat meta-data with the loom files. We used the Python module anndata (v0.7.4) to import the loom files and Seurat meta-data. We extracted $CD4^+$ T cells and $CD8^+$ T cells for the RNA velocity analysis. The UMAP coordinates of $CD4^+$ and $CD8^+$ T cells were mapped to the anndata object, and RNA velocity analysis was performed in "stochastics" mode using Python module scVelo. Finally, the results of the RNA velocity analysis were visualized using the matplotlib (v3.3.1) module. The direction of the arrow indicated the possible future differentiation direction of the cells.

**TCR analysis**

Single-cell V(D)J sequencing data were aligned to the vdj-GRCh38 reference genome using 'cellranger vdj' (v4.0.0). The cellranger vdj pipeline performs V(D)J sequence assembly and paired cell-by-cell clonotype calling. The outputs of cellranger vdj include the productive nucleotide sequences and translated amino acid sequences of the CDR3 region for TCRs (α and β chains). A clonotype was defined as the identical CDR3 sequences of an α-β TCR pair. Cells with the same clonotype were identified as clonal TCRs.

Cells with the same clonotype ID within a $CD4^+$ or $CD8^+$ T cell subtype were

counted for each sample. The percentage of each expanded clonotype (Pexp) was calculated as follows:

$$\text{Pexp}=\frac{\sum_{i=1}^{m} n_i}{N} * 100\%, \text{ in which}$$

m: clonotype ID with attributed cell number ≥ 2;

$n_i$: attributed cell number for clonotype i;

N: total cell number in a subtype for a sample.

The mean Pexp value for each subtype from adjacent normal, OLK and OSCC samples was calculated and used for heatmap plotting.

**Cell-cell interaction analysis**

We analyzed the cell-cell interactions of myeloid cell subsets with $CD4^+$ and $CD8^+$ T cells in adjacent normal, OLK and OSCC tissues, as well as the cell-cell interactions of myofibroblast subsets with $CD4^+$ T cells, $CD8^+$ T cells and macrophages in OSCC tissues, using the Python module cellPhonedb (v.2.0) (18). The Seurat counts file and cell type annotations were input into ‘cellphonedb method statistical_analysis’. The average expression values of a receptor by a cell type and a ligand by another cell type were considered to be the ligand-receptor interaction intensity between the 2 cell types. A null distribution of the mean of the average ligand and receptor expression in the interacting clusters was generated by randomly permuting the cluster labels of all cells with 1000 iterations. The *P* value for the likelihood of cell-type specificity of a given ligand-receptor complex was calculated on the basis of the proportion of the means that were as high as or higher than the actual mean. The significance threshold of cell-cell interactions was *P* value < 0.05. We used the R ggplot2 package (v3.3.2) to visualized

the results.

**Functional enrichment of differentially expressed genes (DEG)**

The enrichment analysis of the DEGs among the neutrophil subsets was performed using the Metascape webtool (www.metascape.org) (19). The gene sets used for the analysis were obtained from the Reactome database. *P*-values are calculated based on the accumulative hypergeometric distribution. Terms with a *P*-value < 0.01 are collected and grouped into clusters based on their membership similarities.

**Gene set variation analysis (GSVA)**

The Gene Set Variation Analysis R package (GSVA, v1.40.1) was applied to identify differentially expressed genes between the 2 myofibroblast subsets (20). Firstly, the gene set scores per cell were calculated for myofibroblasts by GSVA. Subsequently, the significantly enriched gene sets between the 2 myofibroblast subsets were identified and arranged using the R package limma (v3.48.0). The REACTOME gene sets in the R package msigdbr were used for GSVA analysis. Only significant genes (adjusted $P <$ 0.05) were used for further analysis.

**Pseudotime analysis**

The putative differentiation trajectories among ADSCs, fibroblasts and myofibroblasts (ADSC-Fibro-MF subsets) were constructed using the R package Monocle2 (v2.20.0) (21). Firstly, the top 2000 high variable genes (HVGs) in ADSC-Fibro-MFs were extracted using the function FindVariableFeatures in Seurat v3 and set as the ordering genes for ADSC-Fibro-MF subsets. Next, the CellDataSet (CDS) of ADSC-Fibro-MF subsets was constructed using the ordering genes, and the size factors of each cell were

calculated using the estimateSizeFactors and estimateDispersions functions with default parameters. Next, dimension reduction of the CDS was performed using the reduceDimension function with the DDRTree method, in which the size factors and UMI of each cell were normalized by the residualModelFormulaStr algorithm. After dimensionality reduction, the cells were ordered using the orderCells function with default parameters.

**H&E staining, immunohistochemistry and immunofluorescence**

Parts of the dissected murine tongue lesions were harvested and fixed in 10% formalin for 24 hours, followed by sectioning into 4-μm thick slices. The slices were then stained with H&E. The TMAs were a series of tumor samples from 142 OSCC patients, among which one 1.5 mm core of representative region from each tumor sample was selected by two certified pathologists for the construction of TMAs. The TMAs were deparaffinized and rehydrated, after which the samples were permeabilized with 0.2% Triton X-100 and incubated in 3% $H_2O_2$ for 10 minutes. Next, antigen retrieval was performed under high temperature and high pressure for 15 minutes in EDTA buffer. The TMAs were then incubated with rabbit anti-human TDO2 primary antibodies at 4 °C overnight. After washing the TMAs 3 times with PBS, it was incubated with goat anti-rabbit secondary antibodies for 1 hour, stained with DAB for 3 minutes, and finally counterstained with hematoxylin. The images of TMAs were captured and deposited by an Axio Scan.Z1 side scanner (Zeiss). TDO2 expression on TMAs were quantitated using H-score. H-scores were quantified following the method previously described (22). Briefly, the total percentage of TDO2 positive cells and the intensity of the TDO2

staining ($1^+$, $2^+$, or $3^+$), where H-score= (%$1^+$ ×1) + (%$2^+$ ×2) + (%$3^+$ ×3). H-scores range from 0-300, as 0 representing no cell staining with the marker and 300 representing every cell staining with $3^+$. For the cohort in the TMAs, the OSCC patients of TMAs (n =142) were classified equally into TDO2-high (H-scores ≥ 86.5; n=71) and TDO2-low (H-scores < 86.5; n=71) groups based on the median value of H-score.

For immunofluorescence (IF), the samples were incubated with mouse anti-human α-SMA, rabbit anti-human TDO2 or mouse anti-human MCT4 primary antibodies, followed by incubation with goat anti-mouse (dyelight 488) or goat anti-rabbit (dyelight 549) secondary antibodies according to the experimental design. The samples were finally counterstained with DAPI and the results were captured and analyzed by an FV3000 Confocal Laser Scanning Microscope from Olympus Life Science Solutions. The staining and analysis results of the H&E, IHC and IF were checked by 2 certified pathologists.

**Multiplex immunohistochemistry**

For mIHC staining, 4-μm thick FFPE sections of OSCC tissues were stained with the Opal 7-colour fluorescent IHC Kit (PerkinElmer, Massachusetts, USA). First, deparaffinization, rehydration and permeabilization were performed on all slides, followed by 20 minutes of 10% formalin fixation and 15 minutes of Tris-EDTA antigen retrieval under high temperature and high pressure. Afterwards, the slides were incubated with primary antibodies, secondary-HRP antibodies, and Opal TSA dyes for 16 hours (4 °C), 10 minutes (RT) and 20 minutes (RT), respectively. Subsequent rounds of staining consisted of antigen retrieval, primary antibodies, secondary-HRP

antibodies, and Opal TSA dyes. The following proteins were detected with Opal fluorophores: CD8 (opal-690), pan-CK (opal-620), α-SMA (opal-540), TDO2 (opal-520), CD4 (opal-650), Foxp3 (opal-570) PD-1 (opal-570), and TIM3 (opal-650). DAPI was used for nuclear counterstaining. The slides were finally mounted with antifade reagent (AR1109, BOSTER, Wuhan, China). TissueFAXS Imaging software (v7.134) was used to capture the images and identify all markers of interest. Tumor sections from 10 different patients (Pt01_Ca, Pt04_Ca, Pt06_Ca, Pt07_Ca, Pt08_Ca, Pt09_Ca, Pt10_Ca, Pt12_Ca, Pt13_Ca and Pt14_Ca) were stained. The 4 representative fields of the whole-slide scan images (n=10) were selected and quantitatively analyzed by StrataQuest software (TissueGnostics, v7.0.0). For the mIHC staining of murine tumors, the tumors (4MOSC2) were dissected from C57BL/6 mice and repeated the protocols above. A total of 7 tumors (4 in untreated group and 3 in TDO2i group) were used for mIHC staining. The staining protocols were performed as follows: CD8 (opal-690), pan-CK (opal-620), α-SMA (opal-540), TDO2 (opal-520), CD4 (opal-650), Foxp3 (opal-570), GZMB (opal-520) and TIM3 (opal-570). Three to four representative fields from the images were selected for further statistical analysis. The staining and analysis results of the mIHC were also checked by 2 certified pathologists.

**Isolation and culture of myofibroblasts from OSCC**

To isolate primary myofibroblasts from OSCC, OSCC tissues were immersed in PBS with an antibiotic and an antimycotic for 10 minutes. The isolation and culture processes were performed according to previously described protocols (23). In brief, the peripheral or necrotic tissues were removed and the remaining tissues were minced

into pieces with an average volume of 1–2 $mm^3$ using surgical scissors under sterile conditions. Tumor pieces were placed in uncoated plastic tissue culture flasks and allowed to adhere to the bottom for 2–3 minutes. Dulbeccos Modified Eagles Medium (DMEM) with 10% FBS was added to the flasks, after which they were placed in a 5% $CO_2$ incubator at 37 °C. The culture medium was replaced the next day and subsequently changed every 3 days. The myofibroblasts growing from the tumor pieces adhered to the bottom of each flask. After the myofibroblasts covered more than 80% of the bottom of each flask, the flasks were trypsinized gently and the myofibroblasts were transferred to new flasks, in which passaging was continued. The remaining tissue samples were cultured and isolated repeatedly. All myofibroblasts used in the experiments were passaged fewer than 6 times.

## References


1. Stuart T, et al. Comprehensive integration of single-cell data. *Cell.* 2019;177(7):1888-1902.e21.
2. Germain PL, et al. pipeComp, a general framework for the evaluation of computational pipelines, reveals performant single cell RNA-seq preprocessing tools. *Genome Biol.* 2020;21(1):227.
3. Haghverdi L, et al. Batch effects in single-cell RNA-sequencing data are corrected by matching mutual nearest neighbors. *Nat Biotechnol.* 2018;36(5):421-427.
4. Guo X, et al. Global characterization of T cells in non-small-cell lung cancer by single-cell sequencing. *Nat Med.* 2018;24(7):978-985.
5. Cheng S, et al. A pan-cancer single-cell transcriptional atlas of tumor infiltrating myeloid cells. *Cell.* 2021;184(3):792-809.e23.
6. Sathe A, et al. Single-cell genomic characterization reveals the cellular reprogramming of the gastric tumor microenvironment. *Clin Cancer Res.* 2020;26(11):2640-2653.
7. Kalucka J, et al. Single-cell transcriptome atlas of murine endothelial cells. *Cell.* 2020;180(4):764-779.e20.
8. Zilionis R, et al. Single-cell transcriptomics of human and mouse lung cancers reveals conserved myeloid populations across individuals and species. *Immunity.* 2019;50(5):1317-1334.e10.
9. Cillo AR, et al. Immune landscape of viral- and carcinogen-driven head and

neck cancer. *Immunity.* 2020;52(1):183-199.e9.

10. Puram SV, et al. Single-cell transcriptomic analysis of primary and metastatic tumor ecosystems in head and neck cancer. *Cell.* 2017;171(7):1611-1624.e24.
11. Xing X, et al. Decoding the multicellular ecosystem of lung adenocarcinoma manifested as pulmonary subsolid nodules by single-cell RNA sequencing. *Sci Adv.* 2021;7(5):eabd9738.
12. Jin S, et al. Single-cell transcriptomic analysis defines the interplay between tumor cells, viral infection, and the microenvironment in nasopharyngeal carcinoma. *Cell Res.* 2020;30(11):950-965.
13. Kallies A, et al. Precursor exhausted T cells: key to successful immunotherapy? *Nat Rev Immunol.* 2020;20(2):128-136.
14. Liu Y, et al. Tumor-repopulating cells induce PD-1 expression in CD8(+) T cells by transferring kynurenine and AhR activation. *Cancer Cell.* 2018;33(3):480-494.e7.
15. Sadik A, et al. IL4I1 is a metabolic immune checkpoint that activates the AHR and promotes tumor progression. *Cell.* 2020;182(5):1252-1270.e34.
16. Bergen V, et al. Generalizing RNA velocity to transient cell states through dynamical modeling. *Nat Biotechnol.* 2020;38(12):1408-1414.
17. La Manno G, et al. RNA velocity of single cells. *Nature.* 2018;560(7719):494-498.
18. Efremova M, et al. CellPhoneDB: inferring cell-cell communication from combined expression of multi-subunit ligand-receptor complexes. *Nat Protoc.*

2020;15(4):1484-1506.

19. Zhou Y, et al. Metascape provides a biologist-oriented resource for the analysis of systems-level datasets. *Nat Commun.* 2019;10(1):1523.

20. Hanzelmann S, et al. GSVA: gene set variation analysis for microarray and RNA-seq data. *BMC Bioinformatics.* 2013;14:7.

21. Trapnell C, et al. The dynamics and regulators of cell fate decisions are revealed by pseudotemporal ordering of single cells. *Nat Biotechnol.* 2014;32(4):381-386.

22. Finn RS, et al. Estrogen receptor, progesterone receptor, human epidermal growth factor receptor 2 (HER2), and epidermal growth factor receptor expression and benefit from lapatinib in a randomized trial of paclitaxel with lapatinib or placebo as first-line treatment in HER2-negative or unknown metastatic breast cancer. *J Clin Oncol.* 2009;27(24):3908-3915.

23. Wheeler SE, et al. Enhancement of head and neck squamous cell carcinoma proliferation, invasion, and metastasis by tumor-associated fibroblasts in preclinical models. *Head Neck.* 2014;36(3):385-392.
